# Mutation Patterns Define Clinical Heterogeneity in Acute Myeloid Leukemia

**DOI:** 10.7150/jca.135267

**Published:** 2026-08-12

**Authors:** Zhengrong Xu, Ying Zheng, Yi Zheng, Yanyan Yao, Haili Geng, Xiaofan Li, Shao-yuan Wang, Lili Pan

**Affiliations:** 1 Fujian Institute of Hematology, Fujian Provincial Key Laboratory on Hematology, Department of Hematology, Fujian Medical University Union Hospital, Fuzhou 350001, PR China.; 2 Translational Medicine Center on Hematology, Fujian Medical University, Fuzhou 350001, PR China.; 3 Union Clinical Medical Colleges, Fujian Medical University, Fuzhou 350001, PR China.

**Keywords:** acute myeloid leukemia, co-mutations, mutational burden, renal impairment, survival analysis

## Abstract

**Background:**

Acute myeloid leukemia (AML) is a molecularly heterogeneous malignancy where next-generation sequencing (NGS) has revolutionized risk stratification and treatment paradigms. However, the interplay between mutation cooperativity, clinical phenotypes, and biochemical markers of organ dysfunction remains poorly characterized. This study investigates how co-mutational patterns influence hematological/biochemical parameters and survival outcomes in AML.

**Methods:**

In this single-center retrospective study (2017-2024), 1,416 non-M3 AML patients with NGS-confirmed somatic mutations were analyzed. Clinical parameters included hematologic parameters, biochemical markers, and survival outcomes. Multivariate Cox models, Kaplan-Meier analysis, propensity score matching (1:4), and Cohen's d effect sizes were employed to assess mutation-clinical correlations.

**Results:**

A higher mutational burden was associated with older age, elevated platelets, and renal impairment, with *DNMT3A* mutation identified as an independent factor for renal impairment. Multivariable analysis identified higher mutational burden, age, renal impairment, and high white blood cell counts as independent predictors of inferior survival. We identified novel co-occurring (e.g., *KRAS* and *PTPN11*; OR = 3.19; 95% CI, 2.97-3.42) or mutually exclusive (e.g., *CEBPA*-*bZIP* and *NPM1*; OR = 0.03; 95% CI, 0.01-0.17) gene pairs (all adjusted *P* <0.001). The *NPM1*/*IDH1* co-mutation elevated blast percentages in bone marrow and peripheral blood compared to either single mutation alone. Three novel triple-mutations were significantly associated with higher mortality: *IDH2*: *FLT3*-*ITD*: *NPM1* (HR = 4.68), *IDH2*:* SRSF2*:* ASXL1* (HR = 3.31), and *RUNX1*: *TET2*: *NRAS* (HR = 3.08) (all adjusted *P* <0.05).

**Conclusions:**

These findings support integrating genomic and biochemical profiling for AML management, as co-mutation patterns, particularly those affecting renal function, refine risk stratification and highlight the need for tailored monitoring and organ support.

## Introduction

Acute myeloid leukemia (AML) is a highly aggressive and molecularly heterogeneous malignancy originating from the clonal expansion of hematopoietic stem/progenitor cells driven by acquired somatic mutations^1^-^3^. Mutations not only initiate leukemia but also interact synergistically or antagonistically to shape complex clonal architectures, thereby fueling disease progression and therapeutic resistance. For instance, the synergistic effect of *FLT3*-*ITD* and *NPM1c* orchestrates a sophisticated epigenetic collaboration by coordinately remodeling chromatin accessibility, modification, and 3D genome topology^4^. This interconnected rewiring drives leukemogenic transcriptional programs, revealing a sophisticated cooperative regulatory network within the epigenetic regulatory landscape that dictates AML pathogenesis.

Current treatment paradigms—ranging from intensive chemotherapy to targeted therapies and allogeneic stem cell transplantation—rely heavily on molecular-cytogenetic risk stratification to optimize outcomes^5^-^8^. Combinational treatments targeting multiple mutations have gained increasing interest. Treatments with both Wnt/β-catenin inhibitors and *FLT3* inhibitors demonstrate synergistic cytotoxic effects by simultaneously disrupting survival pathways and cooperatively suppressing nuclear β-catenin/c-Myc signaling 9. These findings refine AML precision medicine by integrating co-mutations into clinical management. However, mutation frequency and spectrum in large AML cohorts are rarely reported, hindering customized therapy development. Critical gaps remain in understanding how mutational cooperativity affects outcomes and patient traits, particularly its impact on hematological parameters and organ dysfunction biomarkers. For instance, while *CEBPA-bZIP* mutations are recognized as favorable prognostic in AML^5^, the impacts of their interplay with other mutations on hematological/biochemical parameters remain incompletely characterized. Similarly, the prognostic relevance of mutation patterns in biochemical profiles—particularly renal function-associated metrics such as glomerular filtration rate (GFR) or creatinine clearance rate (Ccr)—has not been systematically explored.

In the retrospective single-center study of 1,416 newly diagnosed AML patients, we combined NGS with hematologic and biochemical profiling to link mutation patterns to phenotypic heterogeneity. We found that specific co-mutations correlate with distinct clinical manifestations, including renal impairment, and should be integrated into risk stratification. These results indicate that combined genomic and biochemical profiling can enhance AML risk assessment and treatment monitoring.

## Methods

### Patients and Samples

**Figure 1:**
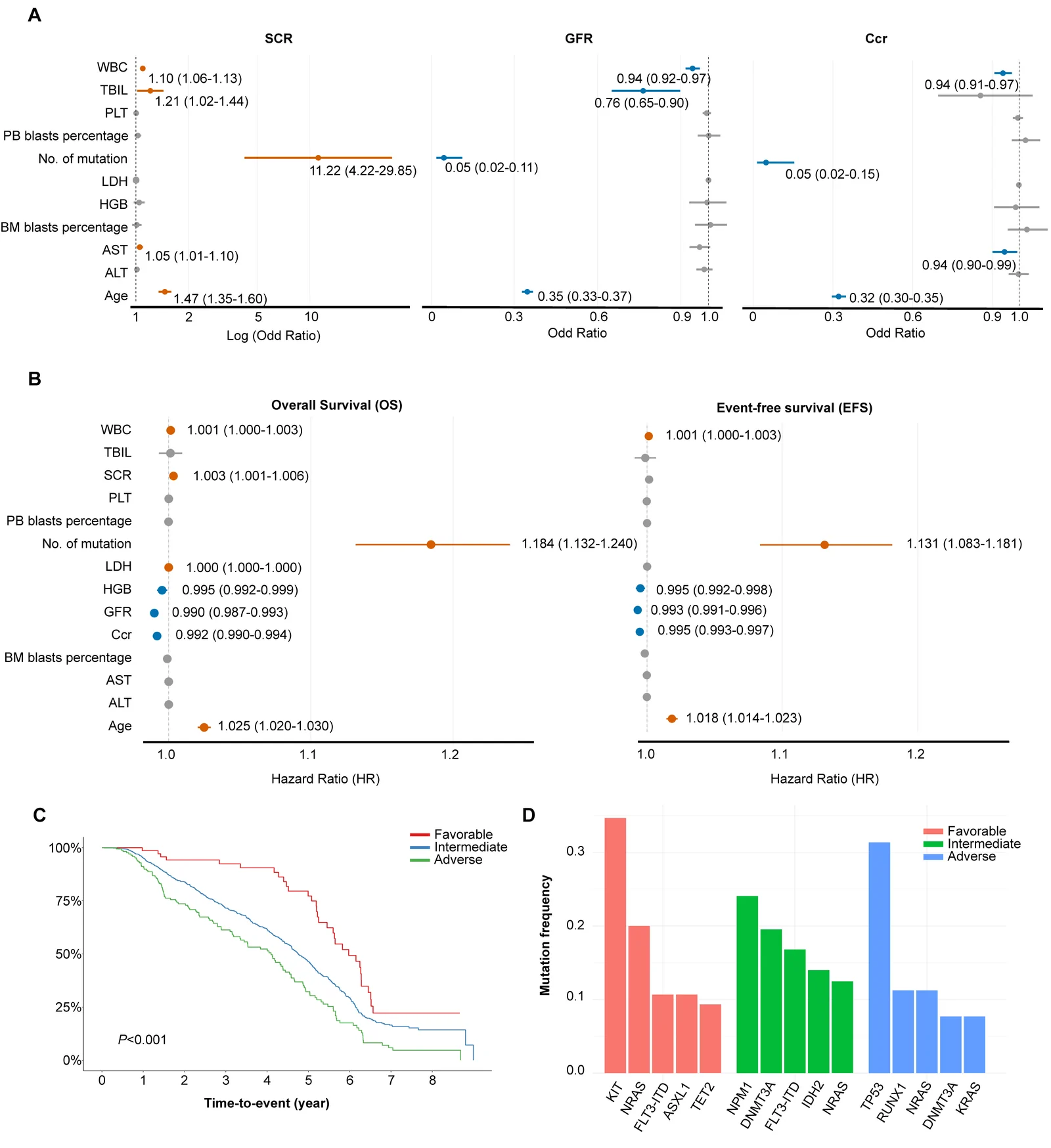
** Associations between baseline characteristics and renal function/survival outcomes. (A)** Logistic regression analysis showing odds ratios (ORs) for the relationship between clinical variables and renal function measures. Orange points represent statistically significant positive associations (*P* <0.05), blue points indicate significant negative associations, and gray points denote non-significant relationships. **(B)** Multi-variate cox proportional hazards model showing hazard ratios (HRs) for the relationship between clinical variables and OS/EFS. **(C)** Kaplan-Meier curves were stratified by prognostic risk groups based on cytogenetic classification according to the latest NCCN guidelines. **(D)** The top five most frequently mutated genes in each prognostic risk category according to NCCN cytogenetic stratification.

**Figure 2:**
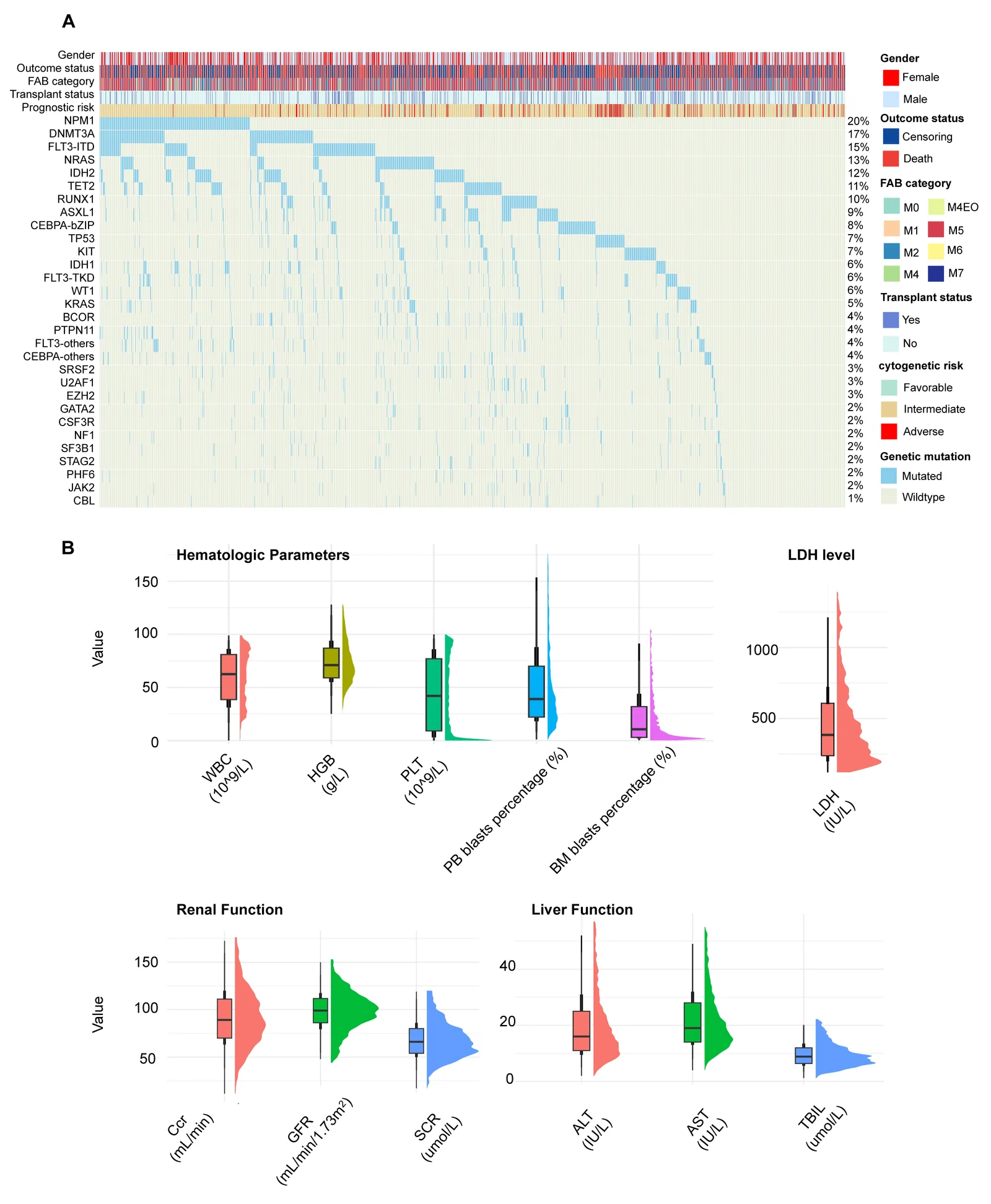
** Mutational landscape and clinical summary of the patient cohort. (A)** Oncoprint of genes with mutation frequencies >1% in our cohort (n = 1,416). Each column represents an individual sample, with vertical line colors indicating mutation status (mutated/unmutated). Gender, outcome, FAB subtype, transplantation status, and NCCN-defined prognostic risk are displayed above the plot. **(B)** Distribution and median values of clinically relevant features in the study population, stratified into four functionally distinct clusters. The Tukey boxplot method (1.5×IQR cutoff) was applied to filter outliers, which were then suppressed in subsequent plots.

This study adhered to the principles of the Declaration of Helsinki and approved by the Expert Committee of Fujian Medical University Union Hospital in China (equivalent to an institutional review board). All participants or their guardians provided written informed consent before enrolment. AML diagnosis was established through a combination of morphology, immunology, cytogenetics and molecular biology classification following the 2008 World Health Organization^10^. This study enrolled 1,416 non-M3 AML patients admitted between 2017 and 2024 (Figure 2A). Among these, VAF data were available for 94 patients (Table S2), and protein mutation site data were available for 683 patients. We calculated Ccr using the gender-specific Cockcroft-Gault equation and GFR using the sex-adjusted CKD-EPI formula. Overall survival (OS) was measured from diagnosis to death or censoring, while event-free survival (EFS) was defined as the time to relapse, death, or censoring. Follow-up data were collected via phone, outpatient, and inpatient electronic records. Cytogenetic risk was stratified per the 2025 NCCN AML guidelines (v2.2025) (Figure 1C).

### Next-Generation Sequencing (NGS)

Peripheral blood of all subjects was collected in EDTA tubes. Genomic DNAs were extracted using QiaAmp Blood DNA Mini kits (Qiagen, CA, USA). The concentration and quality of genomic DNAs were assessed using a NanoDrop® ND-1000 (Thermo Fisher Scientific, MA, USA), the Qubit® 3.0 Fluorometer (Thermo Fisher Scientific, MA, USA) and 1% agarose gel electrophoresis. Paired-end DNA sequencing libraries were prepared through genomic DNA shearing using a Covaris™ (Woburn, MA, USA) sonicator, followed by peak detection, end repair, poly A-tailing, paired-end adaptor ligation, and amplification. The qualified genomic DNA sample was randomly fragmented by Covaris™ (Woburn, MA, USA) and the size of the library fragments is mainly distributed between 250bp and 300bp. End-Repair and A-tailing were then applied to facilitate ligation of the adapters, containing unique barcodes for each sample, specific to the Illumina technology for amplification and sequencing. KAPA Hyper Prep kit was used for these steps, according to the manufacturer's instructions.

Exome capture was performed using Sureselect Human All Exon V6 (Agilent Technologies, Inc., Santa Clara, CA). Each captured library was then loaded onto the NovaSeq6000 platform (Illumina, Inc., San Diego, CA). Each sample was sequenced at the mean depth of 200× to achieve high sensitivity and accuracy for mutations detection.

Target gene capture was performed using SureSelect custom designs (Agilent Technologies, Inc., Santa Clara, CA). Each captured library was loaded onto the nextseq550 platform (Illumina, Inc., San Diego, CA) at the Wuhan Kindstar Global gene Technology (Kindstar, Wuhan, China). The sequencing depth for each sample was maintained at a mean depth of 1000× for high sensitivity and accuracy in mutation detection.

Raw image files were processed using Illumina basecalling Software for base-calling with default parameters, and the sequences of each individual were generated as 150 bp pair-end reads. Pair-end reads were aligned to the human reference genome (GRCh37/HG19) using the Burrows-Wheeler Aligner (BWA, v0.7.17). Strict data quality control was performed in the entire analysis pipeline for clean data, mapping data, variant calling, etc. The Genome Analysis ToolKit (GATK, v4.1.1.0) was used for insertion/deletion realignment, quality score recalibration, and variant identification, with duplicate reads removed by the Picard-tools (v4.1.1.0). ANNOVAR (v201804) was used to annotate mutations. After sequence alignment and variant calling, synonymous variants, intronic variants far away from the exon/intron boundaries, and variants with a minor allelic frequency ≥ 1% in the 1000 Genomes Project, the dbSNP database, and the Exome Aggregation Consortium database were excluded from further analysis. NGS reads were visualized using an Integrated Genomic Viewer. Our screen covered 57 genes implicated in hematologic malignancies, grouped into seven functional clusters (Table S1). Quantitative variant allele frequency (VAF) values were available for a subset of 94 patients. All primary analyses in this study—including associations with clinical parameters, co-mutation patterns, survival models, and propensity score matching—were performed using binary mutation status, which is complete for the entire cohort. VAF data for the 94-patient subset are provided in Table S2 for readers to perform clonal abundance analyses. In addition, recurrent protein hotspot variants were summarized descriptively in patients with available protein-level variant annotation. Variant frequencies were calculated for each protein alteration, and the proportion of each hotspot among all mutations within the corresponding gene was determined.

### Statistical Analysis

All statistical analyses were performed using R 4.4.1. We employed multivariate Cox proportional hazards models adjusted for age, gender, transplant status, prognostic grade^11^, and FAB category to determine the prognostic impact of mutated genes. Univariate survival analyses were conducted using the Kaplan-Meier method, with results visualized via Kaplan-Meier curves and reported with log-rank *p*-values. To ensure balanced and comparable data, we performed propensity score matching (1:4 ratio) based on nearest-neighbor matching for age and gender prior to survival analyses. Additionally, Cohen's d values were calculated to quantify the effect sizes of different mutational patterns on clinical characteristics. To control the false-positive rate, *p*-values were adjusted using the Bonferroni method for analyses requiring stringent control of type I error across multiple comparisons, whereas the Benjamini-Hochberg method was applied in exploratory analyses to control the false discovery rate while maintaining statistical power. Given the limited missingness, we employed the random forest method from multiple imputation approaches to handle the missing data.

## Results

### Mutational Burden Defines Integrated Clinical Characteristics

Table 1 summarizes the baseline characteristics of the 1,416 enrolled patients. The cohort had a sex distribution of 42.5% female and 57.5% male. A higher mutational burden was significantly associated with older age (*P* < 0.001), elevated white blood cell (WBC) and platelet (PLT) counts (*P* < 0.001), an increased blast burden, and impaired renal function, as indicated by elevated serum creatinine (SCR), decreased GFR and Ccr (all *P* < 0.001). Patients with AML-M1 subtype had a significantly higher prevalence of ≥ 3 concurrent mutations compared to other subtypes (52.11% vs. 33.23%, *P* = 0.002). Figure S1C showed that the prognostic impact of mutational burden remained generally consistent across different clinical subgroups.

Multivariate Cox analysis revealed that advanced age, higher mutational burden, and elevated WBC counts were independently associated with impaired renal function (Figure 1A). And these factors together with damaged renal function independently predict poor prognosis in AML patients (Figure 1B).

### Mutation-Specific Organ Toxicity and Prognostic Refinement

The mutation landscape shows genes mutated in > 1% of the cohort (Figure 2A). The most frequently mutated gene in AML was *NPM1* (n = 285, 20%), followed by *DNMT3A* (n = 243, 17%) and *FLT3*-*ITD* (n = 215, 15%) (Figure 2A). To further characterize recurrent mutations within specific genes, we examined the distribution of protein variants in the subset of 683 patients with available protein-level annotation. Consistent with previous findings^12^-^21^, the identified mutations were predominantly located at well-established hotspot residues of AML-associated genes, including NPM1 p.W288Cfs*12, *IDH2 p.R140Q*,* DNMT3A p.R882H*,* NRAS p.G12D*,* FLT3 p.D835Y*,* and KIT p.D816V*. The distribution of these recurrent hotspot variants is summarized in Table S3. Consistent with previous reports^22^-^25^, mutations in *DNMT3A*, *TET2*, *TP53*, *SRSF2*, *RUNX1*, *ASXL1*, and *NPM1* were more prevalent in older patients, whereas *CEBPA*-*bZIP* and *KIT* mutations predominated in younger patients (Figure S2A). Consistent with previous studies, *RUNX1* mutations showed male predominance^26^, ^27^, contrasting with the female predominance of *FLT3*-*ITD* 28. *TP53* (*P* = 0.025) and *CEBPA*-*bZIP* (*P* = 0.022) mutations were significantly more prevalent in males, while *IDH1* was more frequent in females (*P* = 0.014). Nonetheless, *NPM1* was the most frequently mutated gene in both genders (Figure S1A). In our cohort, karyotypes separated patients into three groups with significantly different prognosis (Figure 1C). In line with prior reports^29^, *KIT* mutations were most frequent in patients with favorable-risk cytogenetics (34.67%). The intermediate-risk group was characterized by predominant *NPM1* (19.96%) and *DNMT3A* (15.67%) mutations, whereas the adverse-risk group showed a high prevalence of *TP53* (31.36%) and *RUNX1* (11.24%) mutations (Figure 1D). Analysis of AML subtypes revealed significantly higher frequencies of *NPM1* (35.78%) and *IDH2* (26.61%) mutations in the M0/M1 subtype compared to others (*P* < 0.001 for both; Figure S1B).

Beyond the mutational frequencies across demographic and clinical subgroups, we next investigated the correlations between specific mutations and key laboratory parameters. *TP53*, *BCOR*, *IDH2*, and *U2AF1* mutations correlated with low WBC counts, while *FLT3*-*ITD*, *NPM1*, *NRAS*, *DNMT3A*, and *FLT3*-*others* were associated with high WBC counts^23^, ^30^, ^31^(Figure S2B).* DNMT3A* mutations additionally correlated with elevated PLT counts (Figure S2C). Consistent with previous reports^23^, ^32^-^34^, *TP53* and *ASXL1* mutations showed higher prevalence in patients with low bone marrow (BM) and peripheral blood (PB) blast percentage (Figure S4B-C). *FLT3*-*ITD*, *NPM1*, *DNMT3A*, *IDH1*, and *IDH2* mutations dominated high BM blast groups, while *FLT3*-*ITD*, *NPM1*, and *CEBPA*-*bZIP* mutations characterized high PB blasts percentage groups^23^, ^35^-^38^. Contrary to prior reports indicating no significant correlation between *RUNX1* mutation and BM blast percentage^39^, patients harboring *RUNX1* mutations exhibited reduced BM and PB blasts compared to wild-type cases (Figure S4B-C).

**Figure 3:**
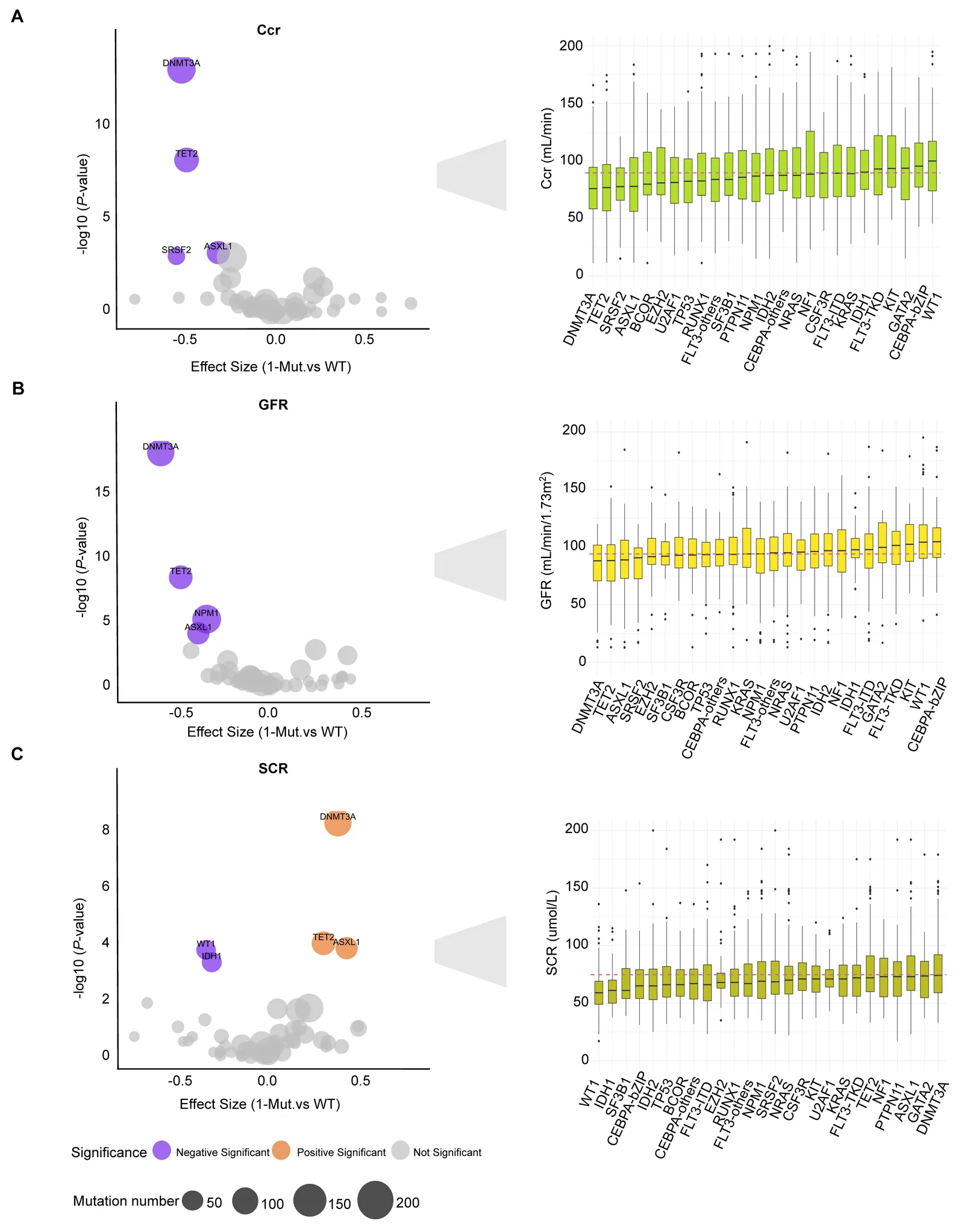
** Association between individual genotypes and renal functions of AML.** Statistical correlation (left) and distribution (right) between renal function indicators (SCR, GFR and Ccr) and individual genotypes for the patients in our cohort. **Left panels**: Orange points indicate statistically significant (Bonferroni FDR <0.05) positive effect sizes while purple points indicate statistically negative effect sizes. Effect sizes (Cohen's d) were calculated between mutated and wild-type patients. *P*-values were calculated using a two-sided Wilcoxon rank sum test. **Right panels**: Red dashed lines denote the mean value for each feature across all patients. For every distribution, boxplots display the first and third quartile boundaries, with a central line marking the median in different gene-mutated patients.

Given limited research on mutations linked to hepatic and renal impairment in AML, we evaluated specific mutation effects. *DNMT3A*, *TET2*, and *ASXL1* mutations correlated with renal impairment (reduced Ccr and GFR, elevated SCR; Figure 3A-C). Renal impairment was defined as elevated SCR or a significant reduction in GFR (*P* < 0.05). Multivariate regression analysis confirmed *DNMT3A* mutations as an independent factor for all renal impairment indicators (GFR, Ccr and SCR), while *TET2* mutations independently affected Ccr and *ASXL1* mutations affected GFR and SCR (Figure S3B). Although the effects of most mutations on liver function are generally marginal, *FLT3*-*ITD* or *NPM1* mutations correlated with elevated aspartate aminotransferase (AST) (Figure S5A), and *CEBPA*-*bZIP* with increased total bilirubin (TBIL) (Figure S5C). Similarly, *IDH2*, *RUNX1*, and *BCOR* mutations correlated with low lactate dehydrogenase (LDH) while *FLT3*-*ITD* and *KIT* mutations associated with high LDH (Figure S3A).

**Figure 4:**
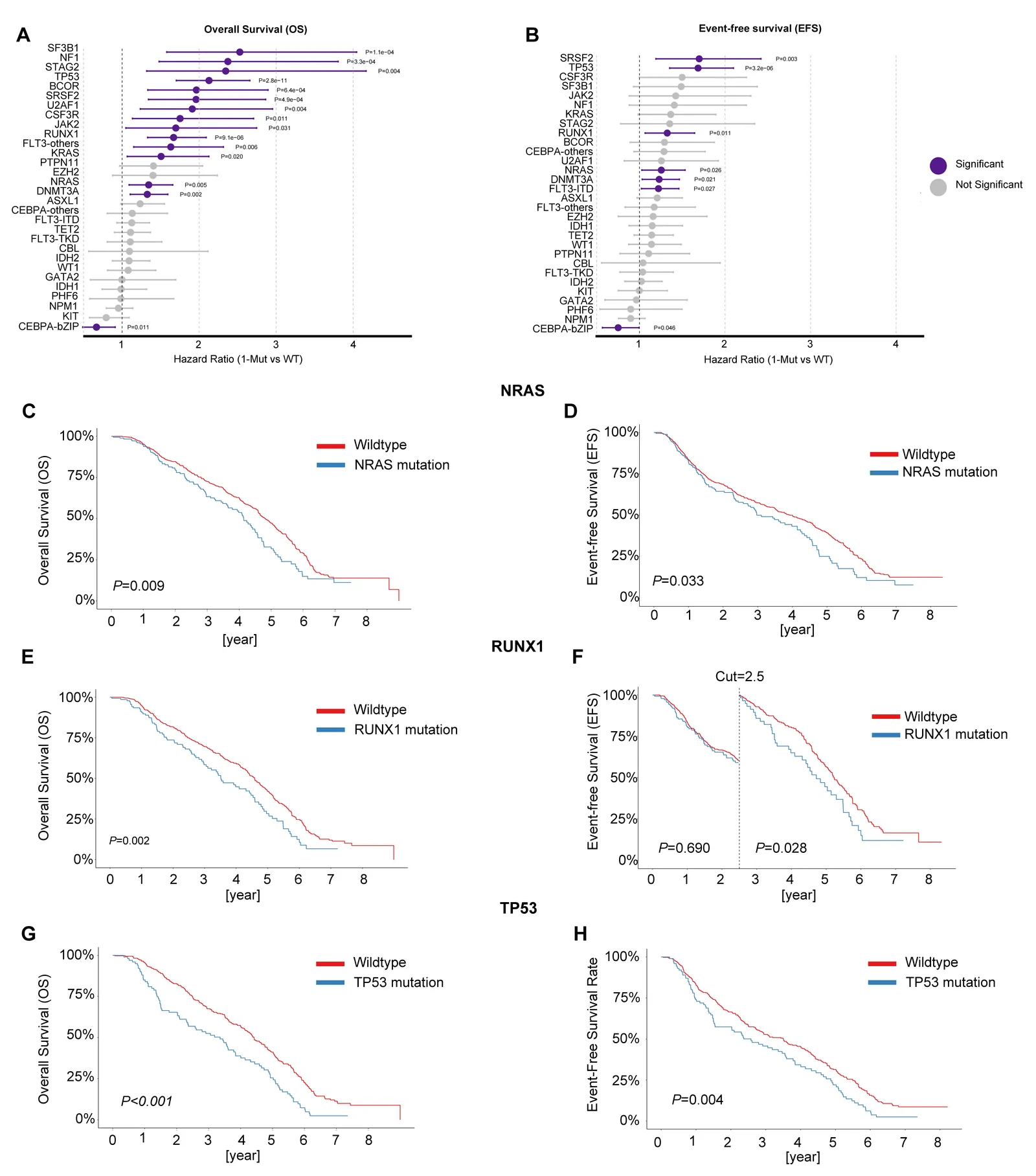
** Association between individual genotypes and survival outcomes of AML. (A)** Forestplot depicting multivariate Cox proportional hazards analysis of overall survival for genes with mutation frequencies >1% in the study cohort, adjusted for age, gender, and transplantation status. Genes demonstrating statistical significance (*P* <0.05) are highlighted in purple. **(B)** Forestplot depicting multivariate Cox proportional hazards analysis of event-free survival (events defined as all-cause death or relapse) for genes with >1% mutation frequency in the study population, adjusted for age, gender, and transplantation status. Genes reaching statistical significance (*P* <0.05) are shown in purple. **(C-H)** Kaplan-Meier survival curves for the mutation of *NRAS*, *RUNX1* and *TP53*. Landmark analysis was applied where curves crossed.

Having established the clinical and laboratory correlates of specific mutations, we next evaluated their prognostic significance through multivariable Cox proportional hazards regression models. Consistent with prior studies^40^-^46^, *RUNX1, DNMT3A*, *SRSF2, NRAS,* and *TP53* mutations were associated with significantly adverse OS and EFS (Figure 4A-H). *CSF3R* mutations predicted adverse OS across all AML subtypes, extending its previously limited prognostic association with *CEBPA*-mutant AML^47^ (Figure S6E). Additionally, we validated the favorable OS (Figure S6J) and EFS (Figure S7C) in AML patients with *CEBPA-bZIP* mutations (HR = 0.67, 95%; CI: 0.49-0.91) (Figure 4A-B). However, *NF1*, *CSF3R, JAK2, KRAS, STAG2, U2AF1, BCOR,* and *FLT3*-*others* only impacted OS without affecting EFS (Figure 4A-B).

### Co-mutation Networks Drive Adverse Outcomes and Organ Toxicity

**Figure 5:**
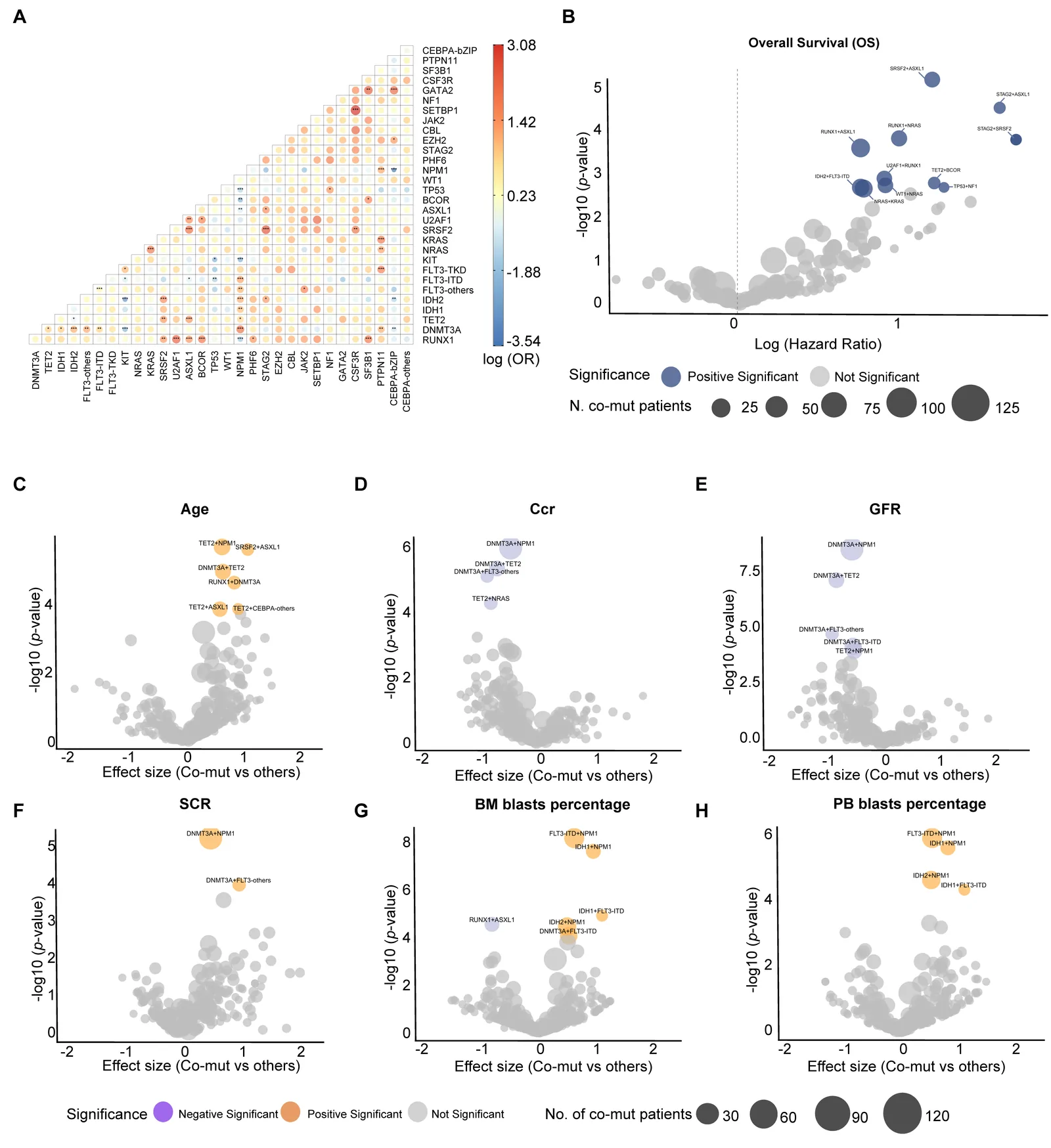
** Distinct patterns of mutation co-occurrence associate with overall survival and clinical characteristics. (A)** Co-occurrence and mutual exclusivity of the most frequent mutations present in de novo AML was performed using a two-sided Fisher's Exact test. Point size and color are based on the magnitude and directionality of the odds ratio based on co-occurrence (red) or mutual exclusivity (blue). Asterisks representing the FDR-corrected significance.** (B)** Summary of pairwise mutations and their association with prognosis based on Cox proportional-hazards regression modeling adjusted for age, gender, and transplantation status. Significant genotypes (Bonferroni FDR <0.05) are colored according to the log-transformed hazard ratio compared to wild-type patients, with purple representing worse prognosis (HR ≥ 1). Points are sized based on the number of patients with co-occurring mutations for each genotype.** (C-H)** Impact of genetic pair mutations on patient clinical characteristics. Orange points indicate statistically significant (Bonferroni FDR <0.05) positive effect sizes while purple points indicate statistically negative effect sizes. Effect sizes (Cohen's d) were calculated between mutated and wild-type patients. *P*-values were calculated using a two-sided Wilcoxon rank sum test.

We comprehensively characterized the co-mutation landscape of recurrently altered genes (mutation frequency > 1%) in the entire cohort. Beyond previously reported associations (e.g., *NPM1*/*PTPN11*, and *SRSF2*/*STAG2*)^23^, we identified several novel co-occurrences (e.g., *KRAS* and *PTPN11*; OR = 3.19; 95% CI, 2.97-3.42; adjusted *P* < 0.001) or mutually exclusive (e.g., *CEBPA*-*bZIP* and *NPM1*; OR = 0.03; 95% CI, 0.01-0.17; adjusted *P* <0.001) gene pairs (Figure 5A; Figure S8A).

Analysis of the association between co-mutation patterns and age revealed that several co-mutations *SRSF2*/*ASXL1*, *TET2*/*NPM1*, and *TET2*/*ASXL1* were enriched in older patients (Figure 5C). Notably, patients with *DNMT3A/NPM1* or *DNMT3A/FLT3-others* co-mutations exhibited significantly elevated SCR, decreased GFR, and reduced Ccr (Figure 5D-F), although these combinations did not confer worsened renal function compared to *DNMT3A* mutations alone. We also assessed the relationship between co-mutation patterns and liver function markers (alanine aminotransferase (ALT), AST, TBIL) and LDH. No co-mutation combinations were significantly associated with liver function parameters (Figure S8C-E).

Consistent with previous study^23^, we validated that *NPM1***/***DNMT3A*, *NPM1***/***FLT3*-*ITD*, and *DNMT3A***/***FLT3*-*ITD* co-mutations were associated with elevated WBC counts, which may contribute to their poor prognosis. Furthermore, *IDH2***/***BCOR* co-mutations were associated with significantly lower WBC counts (Figure S8F). Co-mutations of* NPM1* with *IDH1*, *IDH2*, or *FLT3-ITD* were associated with increased blast proportions (Figure 5G-H). Given that single mutations in these genes were all associated with increased leukemic cell burden, we evaluated whether co-mutations exhibited additive effects relative to single mutations. The *NPM1/IDH1* co-mutation significantly increased BM and PB blast percentages compared to *NPM1* single mutation (β = 16.797 for BM blasts percent, *P* < 0.001; β = 22.101 for PB blast percentages, *P* < 0.001) or *IDH1* single mutation (β = 22.097 for BM blasts percent, *P* < 0.001; β = 35.032 for PB blasts percent, *P* < 0.001). Similarly, *NPM1/FLT3-ITD* co-mutations showed significantly increased blasts compared to* NPM1* single mutation (β = 8.790 for BM blasts percent, *P* = 0.004; β = 10.368 for PB blasts percent, *P* = 0.014), though not compared to *FLT3-ITD* single mutation. Additionally, *NPM1*/*IDH2* co-mutations significantly increased blasts compared to *IDH2* single mutation (β = 9.751 for BM blasts percent, *P* = 0.007; β = 26.648 for PB blasts percent, *P* < 0.001), but not compared to *NPM1* single mutation.

All significant co-occurring mutations were associated with adverse prognosis (Figure 5B). Consistent with prior studies^48^, ^49^, *ASXL1* co-mutations with *RUNX1* (HR = 2.23, adjust *P* < 0.001) or *SRSF2* (HR = 3.27, adjusted *P* < 0.001) were associated with significantly worse outcomes (Figure 5B).

### Specific Triple Mutations Predict Renal Impairment and Poorer Survival

**Figure 6:**
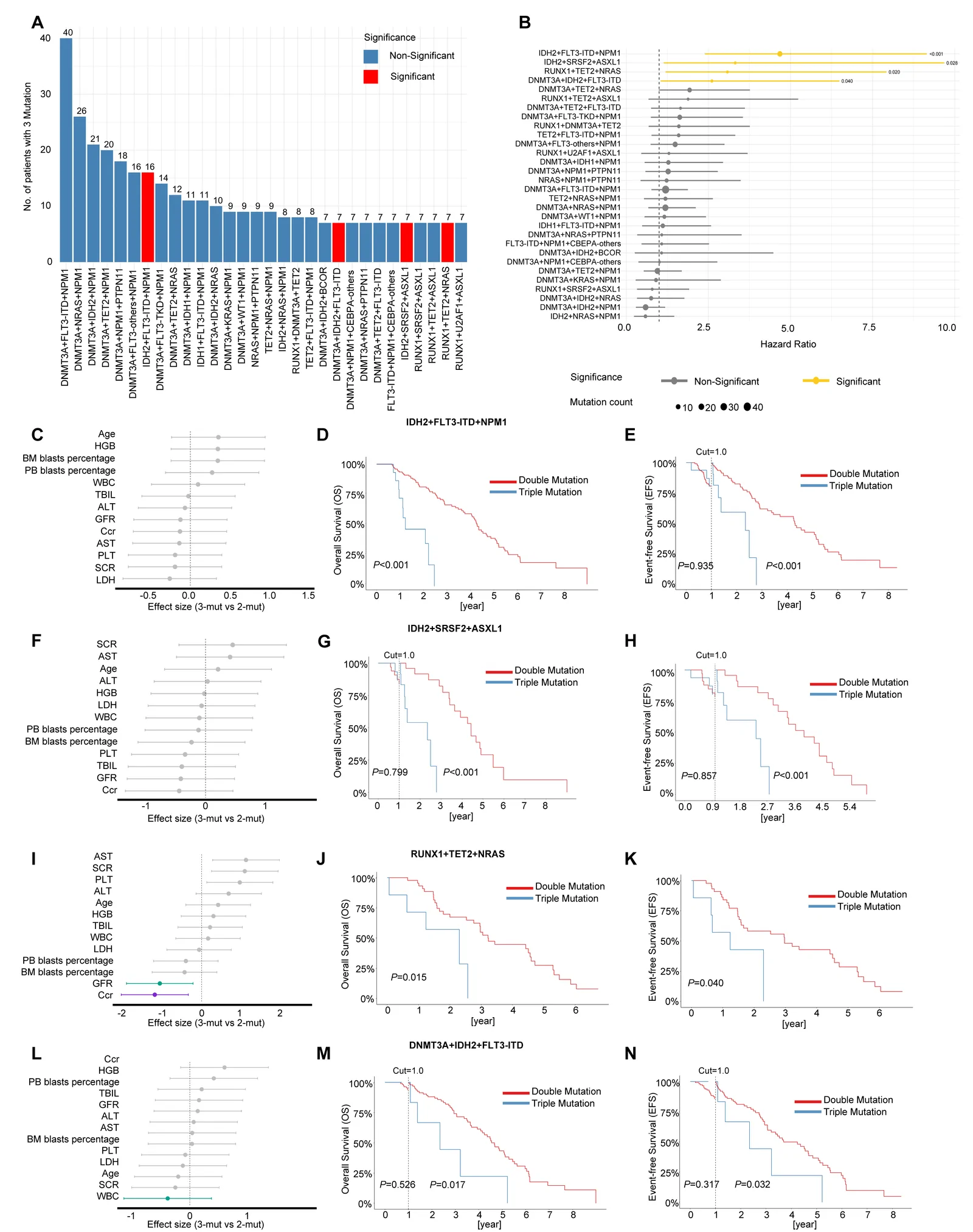
** Mutational landscape of triple mutations and their associations with survival and clinical characteristics. (A)** Frequency distribution of the number of de novo patients with the most frequent 3-way mutation combinations. Bars are colored based on the association with a significant survival correlation (*P* ≤ 0.05) compared to patients with only two genes mutated: red = a significant survival association, gray = no significant association. **(B)** Forestplot depicting survival analysis between triple-mutated and double-mutated genotypes. Points represent the hazard ratios calculated between triple vs. double-mutated patients using a Cox proportional-hazards model, adjusted for age, gender, and transplantation status. Significant genotypes are colored: purple points represent genotypes where all three mutations correlated with worse survival. Points are sized relative to the number of patients with all three mutations and bars represent the 95% confidence intervals of the hazard ratios. **(C-E)** Impact of *IDH2*: *FLT3-ITD*: *NPM1* co-mutation on survival and clinical phenotypes in AML. The point of forestplot is colored based on the level of significance, the purple point indicates genes meeting Bonferroni FDR <0.1, the green point represents those with Bonferroni FDR <0.3, while the gray shows no statistical significance; bars represent the 95% confidence intervals of the effect sizes. Overall survival and event-free survival were depicted using Kaplan-Meier survival curves, with landmark analysis applied at the time point where the curves crossed. **(F-H)** Impact of *IDH2*: *SRSF2*: *ASXL1* co-mutation on survival and clinical phenotypes in AML. **(I-K)** Impact of *RUNX1*: *TET2*: *NRAS* co-mutation on survival and clinical phenotypes in AML. **(L-N)** Impact of *DNMT3A*: *IDH2*: *FLT3-ITD* co-mutation on survival and clinical phenotypes in AML. Overall survival and event-free survival were depicted using Kaplan-Meier survival curves, with landmark analysis applied at the time point where the curves crossed.

We identified 30 distinct triple-mutation genotypes (≥ 7 patients each; Figure 6A). Four combinations exhibited significantly higher co-occurrence frequencies than random expectation: *IDH2*: *FLT3*-*ITD*: *NPM1*, *IDH2*: *SRSF2*: *ASXL1*, *RUNX1*: *TET2*: *NRAS* and *DNMT3A*: *IDH2*: *FLT3*-*ITD* (Figure 6A). We then analyzed the impact of these four triple-mutation combinations on clinical characteristics. We detected the *DNMT3A*:* IDH2*:* FLT3*-*ITD* mutation combination demonstrated significantly reduced WBC counts (Figure 6L). Notably, patients harboring the *RUNX1*:* TET2*:* NRAS* triple mutation exhibited significantly reduced GFR and Ccr (Figure 6I). However, no significant associations were observed between these triple-mutation genotypes and patient age.

In the study by Dunlap et al^50^, compared to *NPM1*-mutated/*FLT3*-*ITD*-negative cases, patients with concurrent *DNMT3A* and *IDH1*/*2* mutations in addition to *NPM1* (triple-positive) exhibited significantly shorter OS. This indicates that these specific co-occurring mutations abrogate the favorable prognosis conferred by isolated *NPM1* mutation. We augmented our analysis to investigate the prognostic association of triple-mutated genotypes compared to pairwise mutated genotypes. Beyond established adverse effects of *NPM1*/*FLT3*-*ITD* co-mutations (per ELN 2022/NCCN 2025 guidelines), the *IDH2*: *FLT3*-*ITD*: *NPM1* triple mutation markedly worsened survival (HR = 4.68, 95% CI = (2.39-9.15), adjusted *P* < 0.001). Consistent with previous report^23^*, DNMT3A*: *IDH2*:* FLT3-ITD* (HR = 2.61, 95% CI = (1.05-6.49) adjusted *P* = 0.040) were associated with worse outcomes (Figure 6B). In addition, we identified another two novel high-risk triple-mutations: *IDH2*:* SRSF2*:* ASXL1* (HR = 3.31, 95% CI = (1.13-9.68), adjusted *P* = 0.028), and *RUNX1*: *TET2*: *NRAS* (HR = 3.08, 95% CI = (1.20-7.92) adjusted *P* = 0.020) (Figure 6B). All significantly reduced OS and EFS (Figure 6D-E, G-H, J-K, M-N). The observed renal impairment in *RUNX1*: *TET2*: *NRAS* triple mutation may contribute to its poor prognosis. The *DNMT3A*: *IDH2*: *FLT3-ITD* mutation combination was associated with leukopenia. It's unknown whether and how reduced WBC counts would impact survival outcome.

## Discussion

Our study demonstrates that high-frequency co-mutation modules (dual/triple-hit) are associated with AML progression, linking mutational accumulation to clonal evolution in 1,416 patients. This moves beyond single-driver genetics. The accumulation of age-related mutations underscores clonal hematopoiesis of indeterminate potential (CHIP) as a leukemogenic precursor^51^. Early recognition of CHIP mutations may enable proactive monitoring for subsequent leukemia driver mutations. Our study suggested the high-risk co-mutations** (**e.g., *ASXL1*-*SRSF2*, *TET2*-*BCOR*) drive leukemogenesis through genetic cooperation and predict poor prognosis. These findings illuminate critical interception points for monitoring CHIP-related genetic evolution and preventing progression to overt leukemia, supporting tailored surveillance strategies.

Our analysis identified four high-risk triple-mutation combinations. Notably, the *RUNX1*:* TET2*:* NRAS* combination was associated with renal dysfunction, which may contribute to its poor outcomes. Despite the conventional association of low WBC with favorable outcomes, the *DNMT3A*: *IDH2*: *FLT3*-*ITD* triple-mutant cohort paradoxically exhibited both reduced leukocyte counts and inferior survival. This apparent contradiction may reflect a stealth aggression phenotype. *IDH2*-derived 2-HG suppresses mitochondrial oxidative phosphorylation^52^, ^53^, diminishing blast proliferation without impairing survival—enabling chemoresistance through stress adaptation. *DNMT3A* mutations drive aberrant extramedullary hematopoiesis and create a permissive epigenetic state, while *FLT3*-*ITD* signaling amplifies proliferative signals^54^, ^55^, facilitating bone marrow niche colonization and extramedullary invasion despite low peripheral blasts.

Beyond the clinical associations described above, the co-occurrence frequencies of these four triple mutation combinations significantly exceeded random expectation, suggesting non-random selective pressures. We propose the following synergistic mechanisms.

For the *IDH2*: *FLT3*-*ITD*:* NPM1* combination, *IDH2*-derived 2-HG may impair dendritic cell differentiation and reduce MHC class II expression, creating an immunosuppressive niche^56^; we hypothesize that this protects *NPM1*-mutant clones, which otherwise elicit neoepitope-specific T-cell responses, from immune elimination^57^. Meanwhile, *FLT3*-*ITD* drives constitutive proliferation and, together with *NPM1* mutation, remodels chromatin accessibility to enforce a differentiation-blocked state^4^, ^58^, ^59^. Thus, immune evasion, proposed protection of an immunogenic clone, and proliferative/differentiation signals represent three complementary hits that no single or double mutation can fully provide.

Turning to *IDH2*: *SRSF2*: *ASXL1*, preclinical evidence shows that Asxl1 truncation plus Srsf2 mutation drives leukemogenesis via immune reprogramming^60^; adding *IDH2* may further disable antigen presentation. The three mutations converge on distinct layers of gene regulation: *IDH2* causes DNA hypermethylation and altered histone methylation^61^-^63^; *SRSF2* disrupts splicing of key hematopoietic regulators^21^; *ASXL1* impairs H3K27 trimethylation^64^. Their co-occurrence likely reflects a need to simultaneously disrupt multiple epigenetic barriers—a task unachievable by any single mutation.

In the *RUNX1*:* TET2*: *NRAS* combination, *RUNX1* is a master regulator of myeloid differentiation^65^. *RUNX1* recruits *TET2* to mediate site-specific DNA demethylation^66^. *TET2* loss compromises DNA methylation fidelity and has been linked to hematopoietic dysfunction^67^-^69^. Their concurrent loss may synergistically disrupt epigenetic regulation, while *NRAS* activation supplies a constitutive proliferative driver. This triad thus may evolve through acquisition of hits that abrogate differentiation (*RUNX1*), epigenetic fidelity (*TET2*), and accelerated proliferation (*NRAS*).

For *DNMT3A*: *IDH2*: *FLT3*-*ITD*, the co-occurrence may follow a combinatorial evolutionary trajectory: *DNMT3A* mutation as an epigenetic primer that alters the methylation landscape^54^; *IDH2* mutation as a metabolic and immune modulator that suppresses oxidative phosphorylation and T-cell function^52^, ^56^; and *FLT3*-*ITD* as a transforming hit that amplifies proliferative signals^59^. This co-occurrence pattern, rather than random co-occurrence, may enable immune evasion, metabolic adaptation, and niche colonization despite low peripheral blast counts.

Collectively, these four combinations illustrate distinct but convergent mechanisms- functional complementation, clonal evolution, and multi-layered epigenetic/immune disruption—that may drive their non-random co-occurrence and adverse prognosis.

A higher mutational burden was associated with impaired renal function indicated by GFR and CCr. Impaired renal function was significantly associated with both single mutations (*DNMT3A*, *TET2*, or *ASXL1*) and, with the *DNMT3A* and *NPM1* co-mutation, suggesting a potential synergistic effect on renal pathology.

We propose a distinct mechanism wherein *DNMT3A* dysfunction induces *PTEN* promoter hypermethylation, suppressing *PTEN* expression and activating the PI3K/AKT pathway^70^, ^71^. This epigenetic dysregulation accelerates renal tubular EMT and TGF-β1-driven fibrotic signaling, exacerbating renal damage^72^.

While *DNMT3A* mutation was independently associated with renal impairment in our multivariable model, we acknowledge that residual confounding by age, sex, and cytogenetic risk may exist. Given the retrospective observational design, this association does not imply causation, and prospective studies are needed to validate this finding.

Collectively, these findings may underscore the need to integrate mutational profiling with renal function monitoring. Prospective validation is warranted to operationalize this precision nephroprotection paradigm.

This study establishes a foundational framework elucidating the genomic-clinical interplay in AML^73^, ^74^. Proactive surveillance of patients with clonal hematopoiesis (CH), coupled with timely intervention of high-risk co-mutations, may facilitate early detection of leukemic transformation^75^. However, this study also has several limitations. Reliable VAF data were available for only 94 of 1,416 patients. Consequently, we were unable to assess the impact of variant allele frequency on prognosis or clonal architecture in the majority of the cohort. While overall sampling was robust, analyses of rare co-mutations or phenotypes were underpowered. Furthermore, the exclusive Chinese cohort enhances homogeneity but may limit global applicability due to known ethnic disparities in AML.

Future studies should develop CH risk models incorporating high-risk co-mutations and renal impairment biomarkers, while optimizing therapies via renal-adapted, mutation-informed drug combinations that avoid nephrotoxicity. Real-time organ surveillance using biomarker panels and toxicity predictors must be implemented. These strategies require validation through multi-center consortia across diverse cohorts to advance precision interception in AML.

## Conclusion

Co-mutation patterns are pivotal in shaping AML phenotypes and outcomes. Our findings highlight the value of integrating genomic and biochemical data, particularly renal function metrics, to improve risk assessment. These findings also support the development of tailored management strategies that address both the leukemia itself and its associated organ toxicities.

## Supplementary Material

Supplementary figures and tables.

## Figures and Tables

**Table 1:**
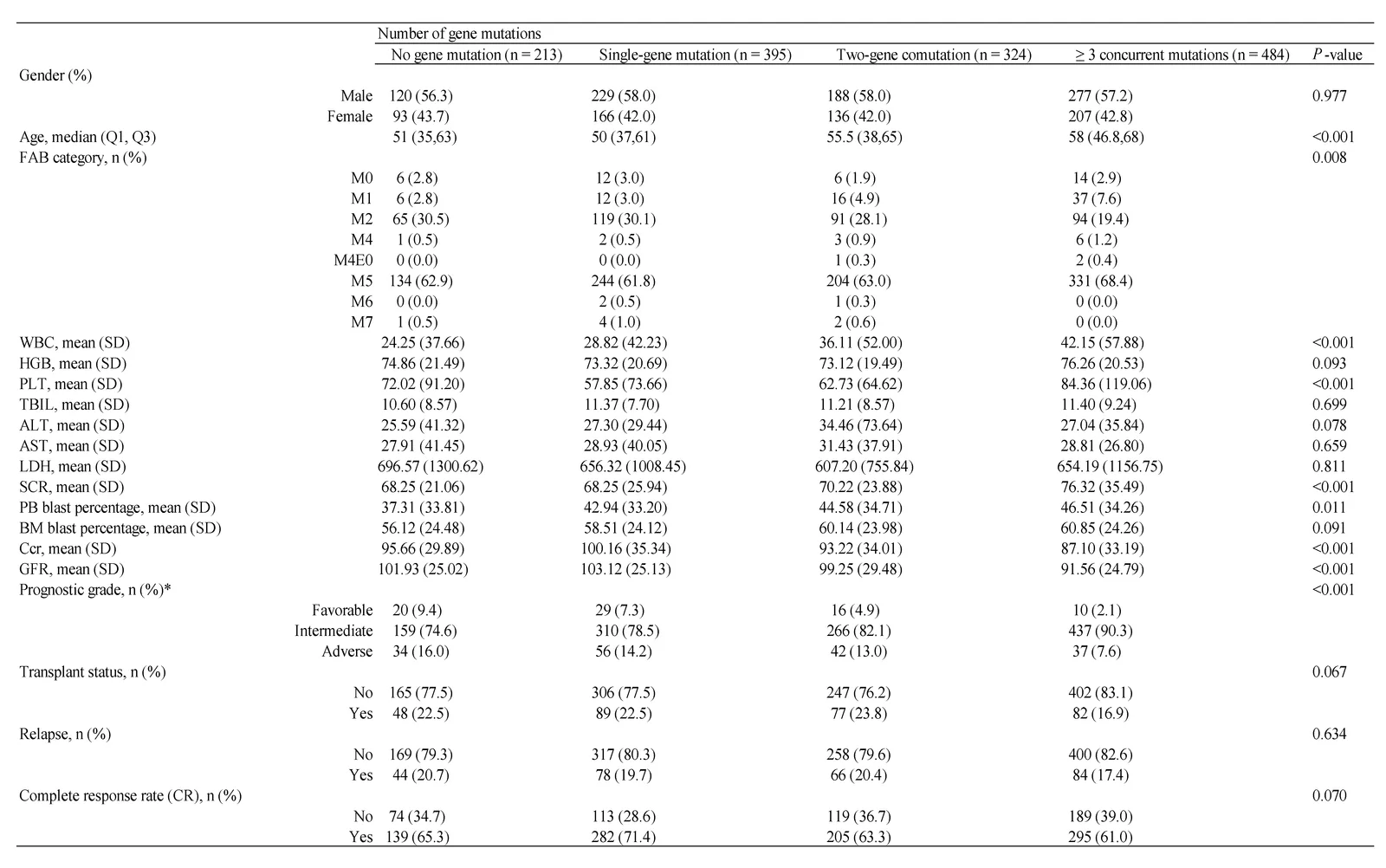
Baseline characteristic.

Data are presented as n (%), median (Q1, Q3), or mean (standard deviation). *The prognostic stratification was performed according to the latest NCCN 2025 guidelines, based on the patient's cytogenetic karyotypes.

## Data Availability

The datasets generated during and/or analyzed during the current study are not publicly available due to ethical restrictions but are available from the corresponding author, Lili Pan (theresalilipan@fjmu.edu.cn), upon reasonable request. The computational code used in this study can also be obtained from the corresponding author upon reasonable request.

## Abbreviations

ALT: alanine aminotransferase
AST: aspartate aminotransferase
BM: bone marrow
Ccr: creatinine clearance rate
GFR: glomerular filtration rate
HGB: hemoglobin
LDH: lactate dehydrogenase
NGS: Next-generation sequencing
PB: peripheral blood
PLT: platelet
SCR: serum creatinine
TBIL: total bilirubin
VAF: variant allele frequency
WBC: white blood cell
