# Supplementary material for: Mutation Patterns Define Clinical Heterogeneity in Acute Myeloid Leukemia
Source: J Cancer. 2026 Aug 12;17(8):1531–45. doi: 10.7150/jca.135267 (PMC13505373; doi:10.7150/jca.135267)
Supplement: Supplementary file 1 [file jcav17p1531s1.pdf]

Supplemental table legend

Supplemental table 1: Gene panel included in NGS analysis and their corresponding functional clusters.

Genes included in the custom NGS panel and their corresponding functional clusters.

Supplemental table 2: Somatic variants identified in 94 patients with available sequencing data, including variant allele frequency (VAF) and predicted protein impact.

Protein changes are described according to Human Genome Variation Society (HGVS) nomenclature. Mutation types were classified as missense, nonsense, frameshift, or splice-site variants based on their predicted effects on protein structure and function. VAF values are reported only for patients with available sequencing data of sufficient depth and quality and were not used as variables in the primary statistical analyses.

Supplemental table 3: Recurrent protein hotspot mutations identified in AML patients with available protein-level variant annotation.

Frequencies represent the number of patients harboring each hotspot variant. Percentages indicate the proportion of each hotspot among all mutations detected within the corresponding gene.

Supplemental figure legend

Supplemental figure 1: Mutation prevalence and differential patterns across gender and AML subtypes.

(A) Mutation profile differences of genes with >5% mutation frequency in both male and female patients. The right heatmap displays sex-specific mutation frequencies. Purple points in the left panel indicate genes with statistically significant differences in mutation rates between genders (Fisher's exact test, P <0.05). **(B)** Mutation frequencies of genes with >5% occurrence across all FAB subtypes. Asterisks denote genes showing significant inter-group differences (Fisher's exact test or Pearson's χ^2 test, *P <0.05, **P <0.01, ***P <0.001). Given the overlapping cytomorphological features between M0 and M1 AML, these subtypes were clustered into a single category. Other rare AML subtypes were grouped as 'Others due to a small sample size. **(C)** Forestplot of subgroup survival analyses evaluating the prognostic impact of mutational burden stratified by clinically relevant variables, including age, gender, ELN risk classification, WBC, GFR, relapse and transplant status. Hazard ratios, 95% confidence intervals, number of events, and P values for interaction are presented for each subgroup.

Supplemental figure 2: Association between individual genotypes and age, WBC and PLT of AML patients.

Left panels: Orange points indicate statistically significant (Bonferroni FDR <0.05) positive

effect sizes while purple points indicate statistically negative effect sizes. Effect sizes (Cohen's d) were calculated between mutated and wild-type patients. *P*-values were calculated using a two-sided Wilcoxon rank sum test. **Right panels**: Red dashed lines denote the mean value for each feature across all mutations. For every distribution, boxplots display the first and third quartile boundaries, with a central line marking the median in both gene-mutated and wild-type patients.

Supplemental figure 3: Association between individual genotypes and LDH, and impact of Specific Gene Mutations on Renal Function Parameters.

(A) Statistical correlation (left) and distribution (right) between LDH and individual genotypes for the patients in our cohort. **(B)** Association of *TET2*, *DNMT3A* and *ASXL1* mutations with renal function parameters (GFR, Ccr and SCR). Beta coefficients were derived from logistic regression analysis, with bars representing 95% confidence intervals.

Supplemental figure 4: Association between individual genotypes and HGB, BM blasts and PB blasts percent of AML patients.

Left panels: Orange points indicate statistically significant (Bonferroni FDR <0.05) positive effect sizes while purple points indicate statistically negative effect sizes. Effect sizes (Cohen's d) were calculated between mutated and wild-type patients. *P*-values were calculated using a two-sided Wilcoxon rank sum test. **Right panels:** Red dashed lines denote the mean value for

each feature across all mutations. For every distribution, boxplots display the first and third quartile boundaries, with a central line marking the median in both gene-mutated and wild-type patients.

Supplemental figure 5: Association between individual genotypes and AST, ALT and TBIL of AML patients.

Left panels: Orange points indicate statistically significant (Bonferroni FDR <0.05) positive effect sizes while purple points indicate statistically negative effect sizes. Effect sizes (Cohen's d) were calculated between mutated and wild-type patients. *P*-values were calculated using a two-sided Wilcoxon rank sum test. **Right panels**: Red dashed lines denote the mean value for each feature across all mutations. For every distribution, boxplots display the first and third quartile boundaries, with a central line marking the median in both gene-mutated and wild-type patients.

Supplemental figure 6: Kaplan-Meier survival curves (Overall survival) for other selected significant genes.

Supplemental figure 7: Kaplan-Meier survival curves (Overall survival and event-free survival) for other selected significant genes.

Supplemental figure 8: Co-mutation patterns and synergistic interactions of gene mutations and their associations with clinical features.

(A) Co-occurrence and mutual exclusivity of mutations present in de novo AML was performed using a two-sided Fisher's Exact test. Significant associations (Bonferroni FDR <0.05) are colored according to the log-transformed odds ratio of co-occurrence (red) or mutual exclusivity (blue). Points are sized based on the number of patients with co-occurring mutations for each genotype. **(B-H)** Impact of genetic pair mutations on other clinical characteristics. Orange points indicate statistically significant (Bonferroni FDR <0.05) positive effect sizes while purple points indicate statistically negative effect sizes. Effect sizes (Cohen' s d) were calculated between mutated and wild-type patients. *P*-values were calculated using a two-sided Wilcoxon rank sum test.

Functional Clusters	Genes
Epigenetic Regulators	DNMT3A, TET2, IDH1, IDH2, ASXL1, ASXL2, EZH2, SETD2, KMT2A, KMT2D, KMT2C
Signal Transduction	FLT3 (ITD, TKD, Others), KIT, NRAS, KRAS, PTPN11, NF1, CBL, JAK1, JAK2, JAK3, MPL, CSF1R, ABL1
Transcription Factors	RUNX1, CEBPA (bZIP, Others), GATA2, WT1, ZBTB7A, AFF1, IKZF1, NOTCH1
Spliceosome	SRSF2, U2AF1, SF3B1
Tumor Suppressors	TP53, PTEN, ATM
Cohesin Complex	STAG2
Others	PHF6, BCOR, FAT1, SETBP1, CRHR1, KRT7, SCYL3, NR1H4, ZMYM4, NPM1, LRBA, G3BP1, ARID1A, CDC45, MYC

Supplemental table 1: Gene panel included in NGS analysis and their corresponding functional clusters. Genes included in the custom NGS panel and their corresponding functional clusters.

Patient ID	Gender	Age	Gene	Protein Change	Mutation Type	Genomic Coordinate	VAF (%)
P01	Male	61	DNMT3A	p.Arg882His	Missense	Chr2:25457242 G>A	38.0
P02	Male	52	NRAS	p.Gly12Ala	Missense	Chr1:115258747 C>G	34.9
			IKZF1	p.Cys95Tyr	Missense	Chr7:50450361 G>A	42.0
			IDH2	p.Arg140Gln	Missense	Chr15:90631934 C>T	32.5
P03	Male	26	CEBPA	p.Gly135fs	Frameshift	Chr19:33792998 delAGTCAAAGTCGCCGCCGCCGCCG	25.3
P04	Male	39	KMT2C	p.Gly315Cys	Missense	Chr7: 151970859 C>A	6.8
P05	Male	36	CEBPA	p.Val314_Leu315insGlyLysGlnArgAsnValGluThrGlnGlnLysVal	Missense	Chr19:33792378 insGGTAAGCAGCGCAACGTGGAGACGCAGCAGAAGGTG	53.0
			CEBPA	p.Ala66Valfs*43	Frameshift	Chr19:33793123 delinsTAGAG	28.7
			GATA2	p.Leu321Pro	Missense	Chr3:128202758 A>G	15.0
			EZH2	p.Lys735Valfs*22	Frameshift	Chr7:148504775 delTC	26.0
P06	Male	23	KIT	p.Asn822Lys	Missense	Chr4:55599340 T>G	26.4
			KMT2D	p.Arg3539Trp	Missense	Chr12:49427975 G>A	8.4
P07	Male	57	DNMT3A	p.Thr386fs	Frameshift	Chr2:25467462insTGCAC	5.8
P08	Female	62	KRAS	p.Gly12Asp	Missense	Chr12:25398284 C>T	22.6
P09	Male	57	EZH2	p.Ala682f	Missense	Chr7:148504781 delCGCC	22.4
P10	Female	53	KIT	p.Asp816Val	Missense	Chr4: 55599321A>T	5.9
			NRAS	p.Gly12Asp	Missense	Chr1:115258747C>T	8.7
P11	Female	27	FLT3	p.Asp835Tyr	Missense	Chr13:28592642C>A	8.8
			IKZF1	p.Gly82Trp	Missense	Chr7:50450321G>T	3.7
			TET2	p.Arg1993Leu	Missense	Chr4:106197645G>T	4.2
			ABL1	p.Lys1029Asn	Missense	Chr9:133760764G>T	2.6
			SRSF2	p.Pro194Gln	Missense	Chr17:74732328G>T	4.1
			SETD2	p.Pro2176Thr	Missense	Chr3:47098616G>T	6.1
			KMT2C	p.Pro2459Gln	Missense	Chr7:151876985G>T	5.8
P12	Male	64	FLT3	p.Ala680Val	Missense	Chr13:28602329 G>A	22.9
			FLT3	p.Asn676Lys	Missense	Chr13:28602340 G>T	13.5
			IDH2	p.Arg172Lys	Missense	Chr15:90631838 C>T	42.6
			NRAS	p.Gly12Asp	Missense	Chr1:115258747 C>T	8.8
			EZH2	p.Arg690Gly	Missense	Chr7:148504758 T>C	88.6
P13	Male	26	FLT3	p.F605delinsGNLKWEF	Missense	Chr13:28608243	43.7
			DCBLD2	p.Glu642Gln	Missense	Chr3:98518620 C>G	38.7
P14	Male	63	PTPN11	p.Glu76Gln	Missense	Chr12:112888210 G>C	22.7
			NRAS	p.Gly13Val	Missense	Chr1:115258744 C>A	9.0

P15	Male	41	FLT3	p.Asp835Tyr	Missense	Chr13:28592642	13.4
			FLT3	p.Trp603delinsTyrGlnTyrGluTyrAspLeuLysTrp	Missense	Chr13:28608248 insATTTGAGATCATATTCATA TTGGT	13.4
P16	Male	34	FLT3	p.Asp835Tyr	Missense	Chr13:28592642 C>A	3.8
			NOTCH1	p.Cys1345Trp	Missense	Chr9:139400313 A>C	14.6
P17	Male	30	DNMT3A	p.Arg882His	Missense	Chr2:25457242 C>T	25.0
			DNMT3A	p.Met548Ile	Missense	Chr2:25467432 C>T	29.5
P18	Male	15	KIT	p.Asn822Lys	Missense	Chr4:55599340 T>G	38.4
			TET2	p.Leu1229Arg	Missense	Chr4:106164818 T>G	2.0
P19	Male	2	SRSF2	p.Pro95_Arg117del	Missense	Chr17:74732896 74732964del	17.4
			KMT2C	p.Arg2610Gln	Missense	Chr7:151874709C>T	44.9
			KMT2C	p.Val2322Ala	Missense	Chr7:151877980A>G	48.5
P20	Male	66	FLT3	p.Asn676Lys	Missense	Chr13:28602340 G>T	37.0
			EZH2	Splicing	Splice-site	Chr7:148529843 C>T	41.6
P21	Male	35	CEBPA	p.Lys304_Gln305insLeu	Missense	Chr19:33792408 G>GCAA	25.5
			CEBPA	p.Asp107Glyfs*56	Frameshift	Chr19:33793001 T>TCGGGAACC	23.3
			GATA2	p.Ala318Thr	Missense	Chr3:128202768 C>T	23.5
			ASXL2	Splicing	Splice-site	Chr2:25967346 C>A	21.0
P22	Female	48	CBL	p.Arg420Gln	Missense	Chr11:119149251 G>A	17.8
P23	Male	54	KIT	p.Asp816Val	Missense	Chr4:55599321 A>T	13.9
P24	Female	55	DNMT3A	p.Arg882His	Missense	Chr2:25457242 C>T	35.4
			FLT3	p.Asn841Thr	Missense	Chr13:28592623 T>G	39.4
P25	Male	56	SF3B1	p.Lys741Asn	Missense	Chr2:198266709 C>G	13.2
P26	Male	57	IDH2	p.Arg140Gln	Missense	Chr15:90631934 C>T	48.9
			DNMT3A	p.Ala410Leufs*7	Frameshift	Chr2:25469529 GCCCCCCAGGGC>G	88.9
			NRAS	p.Gly12Val	Missense	Chr1:115258747 C>A	50.0
			IKZF1	Splicing	Splice-site	Chr7:50367233 G>A	37.0
			IKZF1	p.Thr23Argfs*39	Frameshift	Chr7:50367260 A>AGGTCC	7.6
P27	Male	59	DNMT3A	p.Arg882Cys	Missense	Chr2:25457243 G>A	48.2
P28	Female	64	CEBPA	p.Lys304_Gln305insLeu	Missense	Chr19:33792408 insCAA	42.8
			CEBPA	p.Pro23Argfs*137	Frameshift	Chr19:33793252 delG	41.4
			GATA2	p.Arg362Gln	Missense	Chr3:128200720 C>T	46.7
P29	Male	23	FLT3	p.Glu598_Tyr599insGlyTyrPheTyrValAspPheArgGluTyrGlu	Missense	Chr13:28608261 A>ATTCATATTCTCTGAAATCAACGTAGA AGTACCC	42.1
			NRAS	p.Gly12Asp	Missense	Chr1:115258747 C>T	10.2
			WT1	p.Gln442*	Nonsense	Chr11:32414227 G>A	26.7

P30	Female	55	WT1	p.Glu153*	Nonsense	Chr11:32456435 C>A	16.1
			CEBPA	p.Asn307Lysfs*13	Frameshift	Chr19:33792400 insCGCGT	15.4
			JAK3	p.Glu958dup	Missense	Chr19:17942141-1 794214 3dupCTC	7.7
			JAK3	p.Arg657Gln	Missense	Chr19:17945969 C>T	5.5
			KMT2D	p.Ala2832Val	Missense	Chr12:49432644 G>A	1.8
			KRAS	p.Gly12Asp	Missense	Chr12:25398284 C>T	30.8
			WT1	p.Gln442*	Nonsense	Chr11:32414227 G>A	42.3
			WT1	p.Glu153*	Nonsense	Chr11:32456435 C>A	52.5
P31	Male	52	IDH2	p.Arg172Lys	Missense	Chr15:90631838 C>T	32.9
			ASXL1	p.Gln780*	Nonsense	Chr20:31022853 C>T	28.7
			ZNF135	p.Trp40Arg	Missense	Chr19:58572996 T>A	3.0
P32	Male	67	KIT	p.Lys558Glu	Missense	Chr4:55593606 A>G	19.6
			KIT	p.Asp816Val	Missense	Chr4:55599321 A>T	44.7
P33	Male	48	CEBPA	p.Lys313insGlnLys	Missense	Chr19:33792384 insCTG	48.9
			CEBPA	p.Tyr108fs	Frameshift	Chr19:33792998 insTAGAA	66.6
P34	Female	80	IDH2	p.Arg140Gln	Missense	Chr15:90631934 C>T	41.6
			DNMT3A	Splicing	Splice-site	Chr2:25469028 C>T	38.2
P35	Female	59	RUNX1	p.Arg320*	Nonsense	Chr21:36171607 G>A	42.0
			NRAS	p.Gln61His	Missense	Chr1:115256528 T>A	39.8
			EZH2	p.Cys576Phe	Missense	Chr7:148511175 C>A	41.9
P36	Female	25	CEBPA	p.Gln312dup	Missense	Chr19:33792384 T>TCTG	25.0
			CEBPA	p.Ala72Glyfs*36	Frameshift	Chr19:33793106 G>GC	26.9
P37	Female	35	GATA2	p.Arg398Gln	Missense	Chr3:128200112 C>T	35.0
			GATA2	p.Arg396Trp	Missense	Chr3:128200119 G>A	11.0
P38	Female	26	IDH1	p.Arg132His	Missense	Chr2:209113112 C>T	38.5
			CEBPA	p.Ile68Leufs*41	Frameshift	Chr19:33793119 T>TGTAG	10.4
			PTPN11	p.Glu139Asp	Missense	Chr12:112891083 G>C	6.5
P39	Female	63	FLT3	Splicing	Splice-site	Chr13:28608218 insCAAACTCTAAATTTTCTCTTGGAAACTCCCATTTGAGATCATATT	64.6
P40	Female	13	FLT3	p.836del	Missense	Chr13:28592634	3.5
			FLT3	p.Asp835Val	Missense	Chr13:28592641	13.9
			NRAS	p.Gln61Arg	Missense	Chr1:115256529	11.5
			PTPN11	p.Phe285Ser	Missense	Chr12:112915455	12.4
P41	Female	44	WT1	p.Glu307*	Nonsense	Chr11:32439169 C>A	7.4
			WT1	p.Ala387Valfs*4	Frameshift	Chr11:32417907 G>GCCGA	9.9

P42	Male	26	WT1	p.Leu207Asnfs*49	Frameshift	Chr11:32456274 G>GGCAATGGGTTA	39.0
			NF1	p.Glu2358*	Nonsense	Chr17:29670036 G>T	90.3
P43	Female	53	IDH2	p.Arg140Gln	Missense	Chr15:90631934 C>T	36.5
			KRAS	p.Gly12Asp	Missense	Chr12:25398284 C>T	9.7
			NRAS	p.Gly13Arg	Missense	Chr1:115258745 C>G	19.1
			PTPN11	p.Ser502Ala	Missense	Chr12:112926884 T>G	24.7
P44	Female	51	NRAS	p.Gln61Lys	Missense	Chr1:115256530 G>T	6.2
			NRAS	p.Gln61His	Missense	Chr1:115256528 T>G	27.3
			RNF113B	p.Ala86Val	Missense	Chr13:98829234 G>A	4.0
P45	Female	69	DNMT3A	Splicing	Splice-site	Chr2:25459875 C>T	6.3
			IDH2	p.Arg172Lys	Missense	Chr15:90631838 C>T	12.3
			RUNX1	p.Ser331Leufs*271	Frameshift	Chr21:36164884 T>TGGGGTAA	5.9
			SETD2	p.Ile2499Val	Missense	Chr3:47059166 T>C	11.9
			IKZF1	p.Asp186Tyr	Missense	Chr7:50450372 G>T	8.0
P46	Female	50	KMT2C	p.Ser116*	Nonsense	Chr7:152027728 G>T	39.3
P47	Male	70	FLT3	p.Asp835Leu	Missense	Chr13:28592641 TC>AG	8.8
			IDH1	p.Arg132His	Missense	Chr2:209113112 C>T	47.5
			NRAS	p.Gly13Asp	Missense	Chr1:115258744 C>T	22.6
			NRAS	p.Thr73Ile	Missense	Chr12:112888202 C>T	10.3
			PTPN11	p.Ala461Thr	Missense	Chr12:112926248 G>A	5.3
P48	Male	46	KIT	p.Asp816Tyr	Missense	Chr4:55599320 G>T	21.7
			SETD2	p.Cys1533Ser	Missense	Chr3:4715548247155483delACinsTG	27.2
			SETD2	p.Cys1533Ser	Missense	Chr3:4715548247155483 delACinsTG	27.2
			FLT3	p.Glu77Lys	Missense	Chr13:28636143 C>T	37.0
P49	Male	60	IDH2	p.Arg140Gln	Missense	Chr15:90631934 C>T	12.9
P50	Male	2	CEBPA	p.Ser65Glnfs*43	Frameshift	Chr19:33793130 A>AT	41.0
			CEBPA	p.Arg306_Gln311del	Missense	Chr19:33792386 TGCTGCGTCTCCACGTTGC>T	37.1
			GATA2	p.Leu321Phe	Missense	Chr3:128202759 G>A	15.4
P51	Female	48	FLT3	p.Asp835Tyr	Missense	Chr13:28592642 C>A	30.6
			DNMT3A	p.Tyr533Cys	Missense	Chr2:25467478 T>C	44.2
			PTPN11	p.Phe71Leu	Missense	Chr12:112888197 T>G	11.8
P52	Male	72	CEBPA	p.Arg306_Gln311del	Missense	Chr19:33792386 TGCTGCGTCTCCACGTTGC>T	45.6
			CEBPA	p.Glu50Glyfs*110	Frameshift	Chr19:33793171 CT>C	51.1
			WT1	p.Val376Cysfs*14	Frameshift	Chr11:32417942 A>AG	96.6
			PTPN11	p.Gly60Arg	Missense	Chr12:112888162 G>C	49.9

			IKZF1	p.Arg184Trp	Missense	Chr7:50450366 C>T	41.1
			RNF111	p.Ser48Arg	Missense	Chr15:59323163 A>C	7.7
P53	Female	47	CEBPA	p.Arg306_Gln311del	Missense	Chr19:33792386 TGCTGCGTCTCCACGTTGC>T	42.5
			CEBPA	p.His24Alafs*84	Frameshift	Chr19:33793252 C>CG	41.2
			GATA2	p.Asn317Ile	Missense	Chr3:128202770 T>A	15.9
			CEBPA	p.Arg306_Gln311del	Missense	Chr19:33792386 TGCTGCGTCTCCACGTTGC>T	42.2
			CEBPA	p.His24Alafs*84	Frameshift	Chr19:33793252 C>CG	41.2
			GATA2	p.Asn317Ile	Missense	Chr3:128202770 T>A	15.8
P54	Male	46	DNMT3A	p.Met801Val	Missense	Chr2:25462006 T>C	39.4
			IDH2	p.Arg140Gln	Missense	Chr15:90631934 C>T	46. 1
			IDH2	p.Arg140Trp	Missense	Chr15:90631935 G>A	2.9
			NRAS	p.Gly13Asp	Missense	Chr1:115258744 C>T	31.7
P55	Male	12	KIT	p.Asp816Val	Missense	Chr4:55599321 A>T	8.4
			FLT3	p.Asp835Tyr	Missense	Chr13:28592642 C>A	5.3
			FLT3	p.Asn676Lys	Missense	Chr13:28602340 G>T	10.2
P56	Female	10	NRAS	p.Gly13Asp	Missense	Chr1:115258744 C>T	25.9
			NRAS	p.Gly12Ser	Missense	Chr1:115258748 C>T	12.0
			WT1	p.Ala382Valfs*4	Frameshift	Chr11:32417907 G>GCCGA	10.2
P57	Female	8	FLT3	Splicing	Splice-site	Chr13:28608216 T>TACCAAACTCTAAATTTTCTCTTGGAAACTCCCATTTGAGATCATATTCATATTCTCTGGG	12.5
			FLT3	p.Asp835Tyr	Missense	Chr13:28592642 C>A	5.9
			NRAS	p.Gln61Lys	Missense	Chr1:115256530 G>T	4.2
			KRAS	p.Gly12Asp	Missense	Chr12:25398284 C>T	10.3
			PTPN11	p.Ala72Thr	Missense	Chr12:112888198 G>A	7.0
			TET2	p.Arg1516*	Nonsense	Chr4:106196213 C>T	5.6
P58	Male	27	IDH2	p.Arg140Gln	Missense	Chr15:90631934 C>T	40.8
			FLT3	p.Asp835Tyr	Missense	Chr13:28592642 C>A	14.7
			U2AF1	p.Arg35Leu	Missense	Chr21:44524453 C>A	12.6
			FLT3	p.Asn841Ile	Missense	Chr13:28592623 T>A	4.3
P59	Male	57	KRAS	p.Ala146Thr	Missense	Chr12:25378562 C>T	17.0
			DNMT3A	p.Trp860*	Nonsense	Chr2:25458594 C>T	43.9
			DNMT3A	p.Ser881Arg	Missense	Chr2:25457244 G>C	40.1

P60	Male	75	FLT3	p.Arg595_Glu596insAspGlySerSerAspAsnGluTyrPheTyrValAspPheArg	Missense	Chr13:28608268 T>TTCTCTGAAATCAACGTAGAAGTACTCATTATCTGAGGAGCCA	37.2
P61	Male	35	WT1	p.Ala387Tyrfs*70	Frameshift	Chr11:32417908 C>CCGACCGTA	32.8
P62	Male	68	SRSF2	p.Pro95His	Missense	Chr17:74732959 G>T	44.8
P63	Male	35	KIT	p.Asp816Tyr	Missense	Chr4:55599320 G>T	34.0
P64	Female	44	DNMT3A	p.Arg882His	Missense	Chr2:25457242 C>T	30.6
			RUNX1	p.Met466Leu	Missense	Chr21:36164479 T>A	65.2
			TET2	p.Ala925Cysfs*8	Frameshift	Chr4:106157870 A>AT	37.7
			PTPN11	p.Asp61Gly	Missense	Chr12:112888166 A>G	6.1
			PTPN11	p.Gly503Val	Missense	Chr12:112926888 G>T	12.5
			RUNX1	p.Met466Leu	Missense	Chr21:36164479	49.2
P65	Female	47	IDH2	p.Arg172Lys	Missense	Chr15:90631838 C>T	34.4
P66	Male	74	FLT3	p.Asp835Tyr	Missense	Chr13:28592642 C>A	12.8
			DNMT3A	p.Lys826Met	Missense	Chr2:25459806 T>A	44.5
			TET2	p.Thr229Hisfs*21	Frameshift	Chr4:106155778 GA>G	45.0
			TET2	p.Ile1873Thr	Missense	Chr4:106197285 T>C	50.2
			PTPN11	p.Glu76Gly	Missense	Chr12:112888211 A>G	21.3
P67	Male	45	DNMT3A	p.Ser770Leu	Missense	Chr2:25463184 G>A	38.0
			FLT3	p.Asp835Tyr	Missense	Chr13:28592642 C>A	23.6
			PTPN11	p.Phe285Leu	Missense	Chr12:112910844 T>C	5.0
			PTPN11	p.Gly503Arg	Missense	Chr12:112926887 G>A	6.2
P68	Male	40	DNMT3A	p.Arg882His	Missense	Chr2:25457242 C>T	45.0
			FLT3	p.Tyr597_Glu598insAspPheTyrValAspPheArgGluTyr	Missense	Chr13:28608262 T>TTCATATTCTCTGAAATCAACGTAGA	27.2
			CEBPA	p.Cys56*	Nonsense	Chr19:33793153 G>T	75.0
P69	Male	63	RUNX1	p.Gly199Arg	Missense	Chr21:36231789 C>T	71.4
			FLT3	p.Asn841Ile	Missense	Chr13:28592623 T>A	32.8
P70	Female	31	KIT	p.Asp816His	Missense	Chr4:55599320 G>C	16.3
			KRAS	p.Gly13Asp	Missense	Chr12:25398281 C>T	13.4
P71	Female	54	DNMT3A	p.Arg882His	Missense	Chr2:25457242 C>T	35.5
			FLT3	p.Tyr597_Glu598insAspTyrValAspPheArgGluTyr	Missense	Chr13:28608263 _28608286dup	25.4
P72	Male	44	NRAS	p.Gly13Cys	Missense	Chr1:115258745 C>A	42.4
P73	Male	59	CEBPA	p.Gln83Serfs*77	Frameshift	Chr19:33793073 TG>T	76.2
P74	Female	74	DNMT3A	p.Phe752del	Missense	Chr2:25463235 CAGA>C	45.5
			TET2	p.Asn1118Ilefs*19	Frameshift	Chr4:106158446 TA>T	57.1
			WT1	p.Arg380Metfs*5	Frameshift	Chr11:32417914 C>AT	45.8

			CEBPA	p.Met218Profs*102	Frameshift	Chr19:33792673 G>GGGGTA	51.3
			FLT3	p.Val491Leu	Missense	Chr13:28609758 C>G	40.8
P75	Male	33	FLT3	p.Asp835Tyr	Missense	Chr13:28592642 C>A	40.7
P76	Female	66	DNMT3A	p.Gln656*	Nonsense	Chr2:25464547 G>A	38.0
			DNMT3A	p.Ile661Ser	Missense	Chr2:25464531 A>C	47.2
			FLT3	p.Asp835His	Missense	Chr13:28592642 C>G	7.0
			NRAS	p.Gly12Ser	Missense	Chr1:115258748 C>T	10.2
			PTPN11	p.Ala72Thr	Missense	Chr12:112888198 G>A	8.1
			RUNX1T1	p.Val144Phe	Missense	Chr8:93023247 C>A	35.3
			TET2	p.Arg1302Gly	Missense	Chr4:106180876 A>G	38.2
P77	Female	66	RUNX1	p.Asp198Asn	Missense	Chr21:36231792 C>T	41.8
			KIT	p.Asp816Val	Missense	Chr4:55599321 A>T	39.2
			U2AF1	p.Ser34Phe	Missense	Chr21:44524456 G>A	49.9
			RUNX1	p.Asp198Asn	Missense	Chr17:41256275	48.2
P78	Male	49	FLT3	p.Tyr599_Glu608dup	Missense	Chr13:28608231 T>TTTCTCTTGGAAACTCCCATTTGAGATCATA	37.9
P79	Female	36	IDH2	p.Arg140Gln	Missense	Chr15:90631934 C>T	44.8
			DNMT3A	p.Trp440*	Nonsense	Chr2:25469138 C>T	39.4
			NRAS	p.Gly12Cys	Missense	Chr1:115258748 C>A	41.0
			NOTCH1	p.Phe1592_Leu1593insGly	Missense	Chr9:139399366 G>GCCC	27.7
			NOTCH1	p.Phe1592delinsLeuSer	Missense	Chr9:139399367 G>GGAT	16.1
P80	Female	20	ASXL1	p.Ile641Hisfs*17	Frameshift	Chr20:31022433 G>GC	44.2
			EZH2	p.Lys498Glufs*23	Frameshift	Chr7:148513790 C>CT	37.9
			EZH2	p.Gly159Arg	Missense	Chr7:148526829 C>T	34.9
			NOTCH1	p.Gln2381*	Nonsense	Chr9:139391050 G>A	52.8
P81	Male	2	JAK2	p.Val617Phe	Missense	Chr9:5073770 G>T	5.4
			MPL	p.Ser505Asn	Missense	Chr1:43814979 G>A	9.5
P82	Male	65	IDH2	p.Arg172Lys	Missense	Chr15:90631838 C>T	27.1
			KRAS	p.Gly12Arg	Missense	Chr12:25398285 C>G	19.3
			WT1	p.Gln281*	Nonsense	Chr11:32449533 G>A	42.2
			ZNF114	p.Ala318Thr	Missense	Chr19:48789695 G>A	21.8
P83	Male	30	KRAS	p.Gly12Ala	Missense	Chr12:25398284 C>G	27.3
			NRAS	p.Gly12Ala	Missense	Chr1:115258747 C>G	8.5
			DNMT3A	Splicing	Splice-site	Chr2:25459876 T>A	32.3
			DNMT3A	Splicing	Splice-site	Chr2:25464428 T>C	38.1
P84	Female	24	FLT3	p.Asp835Tyr	Missense	Chr13:28592642 C>A	6.5

			NRAS	p.Gly12Asp	Missense	Chr1:115258747 C>T	6.7
			KRAS	p.Gly12Asp	Missense	Chr12:25398284 C>T	20.5
P85	Male	16	NF1	p.Arg192Gln	Missense	Chr17:29497004 G>A	17.6
P86	Female	41	TET2	p.Arg814Cys	Missense	Chr4:106157539 C>T	50.7
P87	Male	30	KRAS	p.Gly12Ala	Missense	Chr12:25398284 C>G	27.3
			NRAS	p.Gly12Ala	Missense	Chr1:115258747 C>G	8.5
P88	Female	41	AFF1	p.Leu836Gln	Missense	Chr4:88046229 T>A	67.8
P89	Male	23	KIT	p.Asp816His	Missense	Chr4:55599320 G>C	38.2
P90	Female	41	DNMT3A	p.Arg635Trp	Missense	Chr2:25466800 G>A	34.6
			DNMT3A	p.Gln249*	Nonsense	Chr2:25471016 G>A	34.2
			IDH1	p.Arg132Gly	Missense	Chr2:209113113 G>C	32.0
P91	Female	64	EZH2	p.Arg313Leu	Missense	Chr7:148516749 C>A	6.1
			FLT3	p.Phe612_Gly613insArgSerPheArgGluTyrGluTyrAspLeuLysTrpGluPheProArgGluAsnLeuGluPhe	Missense	Chr13:28608220 A>AAACTCTAAATTTTCTCTTGGAAACTCCCATTTGAGATCATATTCATATTCTCTGAAACTCCTG	40.7
			EZH2	p.Arg313Leu	Missense	Chr7:148516749 C>A	6.1
P92	Male	64	FLT3	p.Asp835Tyr	Missense	Chr13:28592642 C>A	7.5
			IDH1	p.Arg132His	Missense	Chr2:209113112 C>T	21.4
			KMT2D	p.Ser3890Argfs*85	Frameshift	Chr12:49426805 CCATGGGCTGAGCG>C	15.0
P93	Female	72	IDH1	p.Arg132Cys	Missense	Chr2:209113113 G>A	33.2
			RUNX1	p.Ser373Leufs*227	Frameshift	Chr21:36164759 G>GC	29.6
			KMT2A	p.Glu1444Aspfs*17	Frameshift	Chr11:118355689 AG>A	6.7
P94	Male	66	IDH1	p.Arg132Cys	Missense	Chr2:209113113 G>A	40.0
			SRSF2	p.Pro95_Arg102del	Missense	Chr17:74732935 CGGCGGCTGTGGTGTGAGTCCGGGG>C	47.7
			TET2	p.Gln904*	Nonsense	Chr4:10615780 9 C>T	1.7

Supplemental table 2: Somatic variants identified in 94 patients with available sequencing data, including variant allele frequency (VAF) and predicted protein impact. Protein changes are described according to Human Genome Variation Society (HGVS) nomenclature. Mutation types were classified as missense, nonsense, frameshift, or splice-site variants based on their predicted effects on protein structure and function. VAF values are reported only for patients with available sequencing data of sufficient depth and quality and were not used as variables in the primary statistical analyses.

Gene	Protein Variant	No. of Patients	Total Patients with Gene Mutation	Percentage of Gene Mutations (%)
NPM1	p.W288Cfs*12	113	144	78.5
IDH2	p.R140Q	77	103	74.8
DNMT3A	p.R882H	58	138	42
NRAS	p.G12D	34	139	24.5
FLT3	p.D835Y	32	109	29.4
FLT3	ITD	19	109	17.4
IDH1	p.R132C	27	57	47.4
KIT	p.N822K	22	76	28.9
SRSF2	p.P95H	16	28	57.1
KRAS	p.G12D	14	37	37.8
ASXL1	p.G646Wfs*12	13	66	19.7
JAK2	p.V617F	13	15	86.7
U2AF1	p.S34F	12	23	52.2
CEBPA	p.H24Afs*84	9	173	5.2
CSF3R	p.T618I	8	18	44.4
SF3B1	p.K700E	8	17	47.1
TET2	p.R814C	6	112	5.4
WT1	p.A382Vfs*4	6	56	10.7
PTPN11	p.A72T	4	45	8.9
RUNX1	p.W106C	4	90	4.4

Supplemental table 3: Recurrent protein hotspot mutations identified in AML patients with available protein-level variant annotation. Frequencies represent the number of patients harboring each hotspot variant. Percentages indicate the proportion of each hotspot among all mutations detected within the corresponding gene.

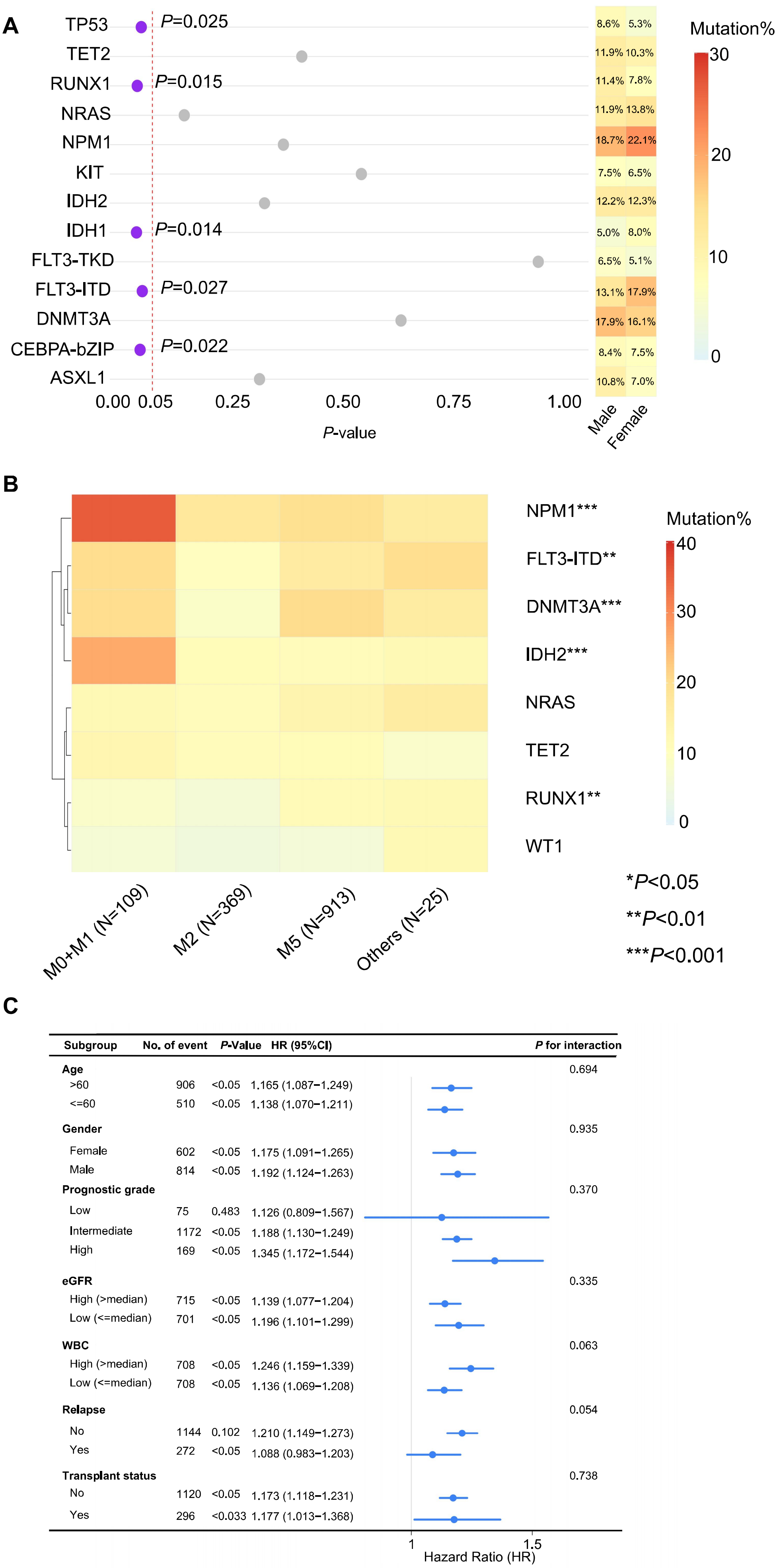
A
TP53
TET2
RUNX1
NRAS
NPM1
KIT
IDH2
IDH1
FLT3-TKD
FLT3-ITD
DNMT3A
CEBPA-bZIP
ASXL1
P=0.025
P=0.015
P=0.014
P=0.027
P=0.022
0.00 0.05 0.25 0.50 0.75 1.00
P-value
8.6% 5.3%
11.9% 10.3%
11.4% 7.8%
11.9% 13.8%
18.7% 22.1%
7.5% 6.5%
12.2% 12.3%
5.0% 8.0%
6.5% 5.1%
13.1% 17.9%
17.9% 16.1%
8.4% 7.5%
10.8% 7.0%
Male
Female
Mutation%
30
20
10
0
B
NPM1***
FLT3-ITD**
DNMT3A***
IDH2***
NRAS
TET2
RUNX1**
WT1
M0+M1 (N=109)
M2 (N=369)
M5 (N=913)
Others (N=25)
Mutation%
40
30
20
10
0
*P<0.05
**P<0.01
***P<0.001
C
Subgroup No. of event P-Value HR (95%CI) P for interaction
Age 0.694
>60 906 <0.05 1.165 (1.087−1.249)
<=60 510 <0.05 1.138 (1.070−1.211)
Gender 0.935
Female 602 <0.05 1.175 (1.091−1.265)
Male 814 <0.05 1.192 (1.124−1.263)
Prognostic grade 0.370
Low 75 0.483 1.126 (0.809−1.567)
Intermediate 1172 <0.05 1.188 (1.130−1.249)
High 169 <0.05 1.345 (1.172−1.544)
eGFR 0.335
High (>median) 715 <0.05 1.139 (1.077−1.204)
Low (<=median) 701 <0.05 1.196 (1.101−1.299)
WBC 0.063
High (>median) 708 <0.05 1.246 (1.159−1.339)
Low (<=median) 708 <0.05 1.136 (1.069−1.208)
Relapse 0.054
No 1144 0.102 1.210 (1.149−1.273)
Yes 272 <0.05 1.088 (0.983−1.203)
Transplant status 0.738
No 1120 <0.05 1.173 (1.118−1.231)
Yes 296 <0.033 1.177 (1.013−1.368)
1 1.5
Hazard Ratio (HR)

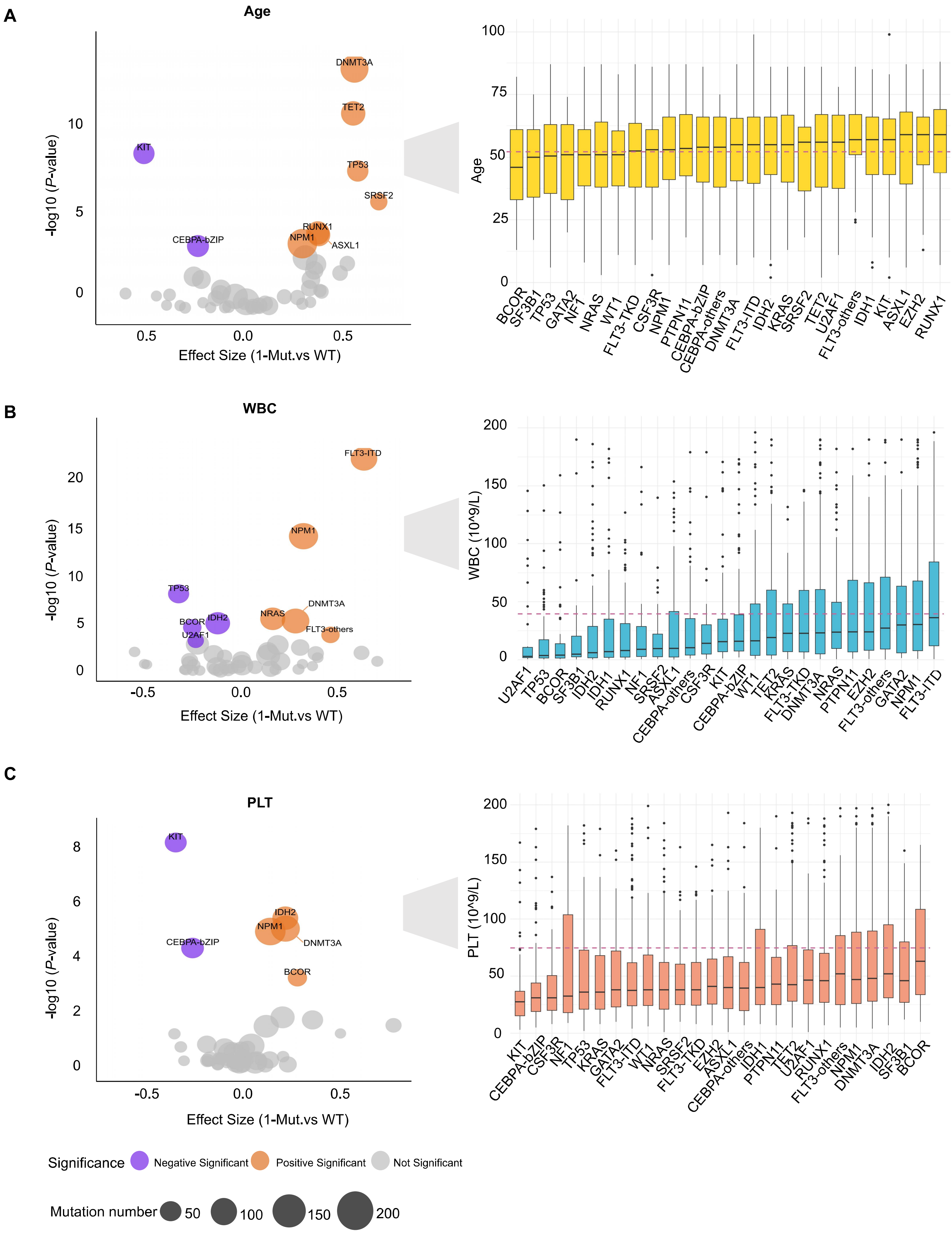
A
Age
DNMT3A
TET2
KIT
TP53
SRSF2
RUNX1
CEBPA-bZIP
NPM1
ASXL1
-log10 (P-value)
Effect Size (1-Mut.vs WT)
100
75
50
25
0
Age
BCOR
SF3B1
TP53
GATA2
NF1
NRAS
WT1
FLT3-TKD
CSF3R
NPM1
PTPN11
CEBPA-bZIP
CEBPA-others
DNMT3A
FLT3-ITD
IDH2
KRAS
SRSF2
TET2
U2AF1
FLT3-others
IDH1
KIT
ASXL1
EZH2
RUNX1
B
WBC
FLT3-ITD
NPM1
TP53
DNMT3A
NRAS
IDH2
BCOR
FLT3-others
U2AF1
-log10 (P-value)
Effect Size (1-Mut.vs WT)
WBC (10^9/L)
U2AF1
TP53
BCOR
SF3B1
IDH2
IDH1
RUNX1
NF1
SRSF2
ASXL1
CEBPA-others
CSF3R
KIT
CEBPA-bZIP
WT1
TET2
KRAS
FLT3-TKD
DNMT3A
NRAS
PTPN11
EZH2
FLT3-others
GATA2
NPM1
FLT3-ITD
C
PLT
KIT
IDH2
NPM1
CEBPA-bZIP
DNMT3A
BCOR
-log10 (P-value)
Effect Size (1-Mut.vs WT)
PLT (10^9/L)
KIT
CEBPA-bZIP
CSF3R
NF1
TP53
KRAS
GATA2
FLT3-ITD
WT1
NRAS
SRSF2
FLT3-TKD
EZH2
ASXL1
CEBPA-others
IDH1
PTPN11
TET2
U2AF1
RUNX1
FLT3-others
NPM1
DNMT3A
IDH2
SF3B1
BCOR
Significance
Negative Significant
Positive Significant
Not Significant
Mutation number
50
100
150
200

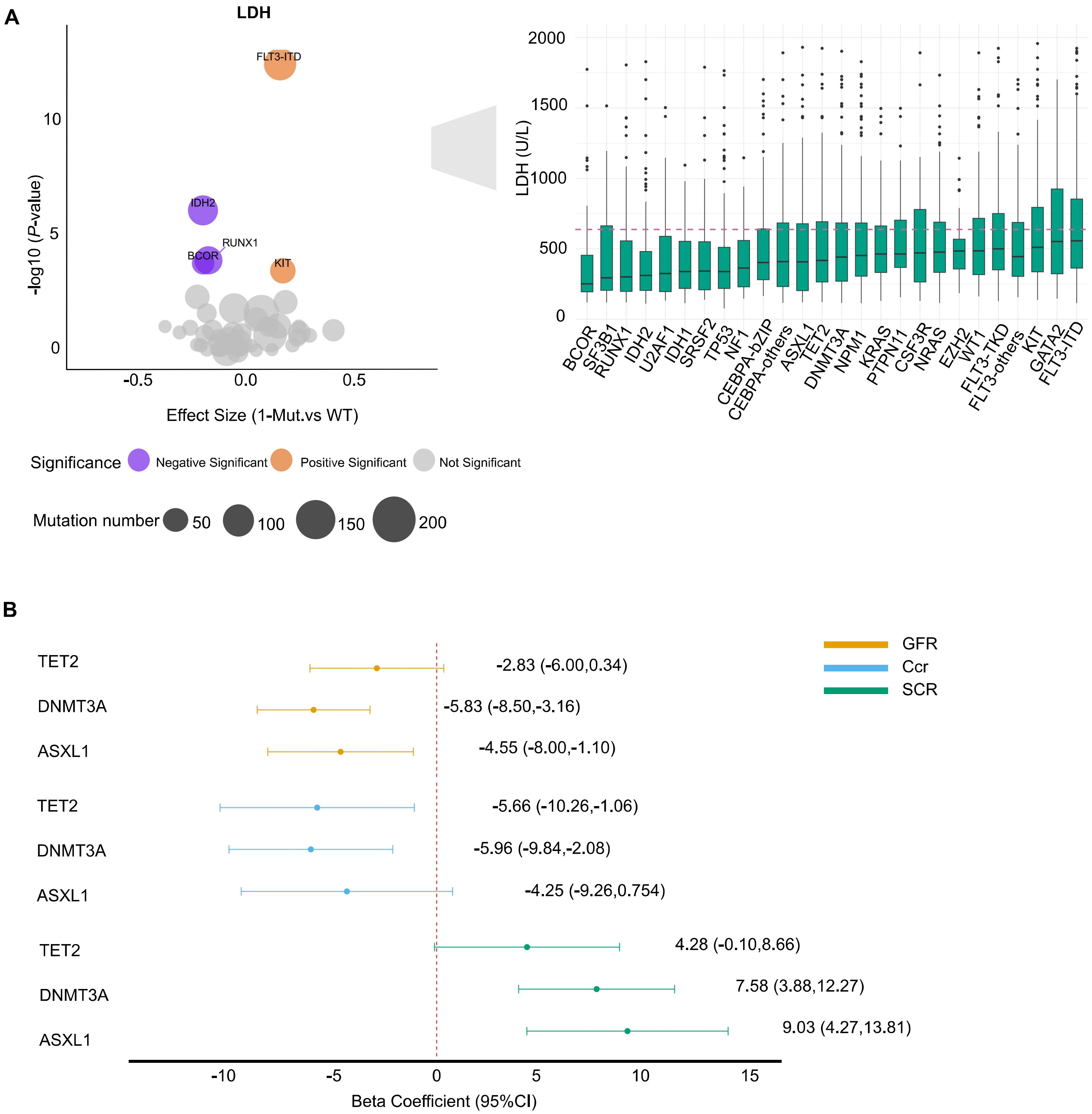

A
LDH
FLT3-ITD
IDH2
RUNX1
BCOR
KIT
-log10 (P-value)
Effect Size (1-Mut.vs WT)
-0.5
0.0
0.5
0
5
10
Significance
Negative Significant
Positive Significant
Not Significant
Mutation number
50
100
150
200
LDH (U/L)
0
500
1000
1500
2000
BCOR SF3B1 RUNX1 IDH2 U2AF1 IDH1 SRSF2 TP53 NF1 CEBPA-bZIP CEBPA-others ASXL1 TET2 DNMT3A NPM1 KRAS PTPN11 CSF3R NRAS EZH2 WT1 FLT3-TKD FLT3-others KIT GATA2 FLT3-ITD
B
GFR
Ccr
SCR
TET2
DNMT3A
ASXL1
TET2
DNMT3A
ASXL1
TET2
DNMT3A
ASXL1
-2.83 (-6.00,0.34)
-5.83 (-8.50,-3.16)
-4.55 (-8.00,-1.10)
-5.66 (-10.26,-1.06)
-5.96 (-9.84,-2.08)
-4.25 (-9.26,0.754)
4.28 (-0.10,8.66)
7.58 (3.88,12.27)
9.03 (4.27,13.81)
-10
-5
0
5
10
15
Beta Coefficient (95%CI)

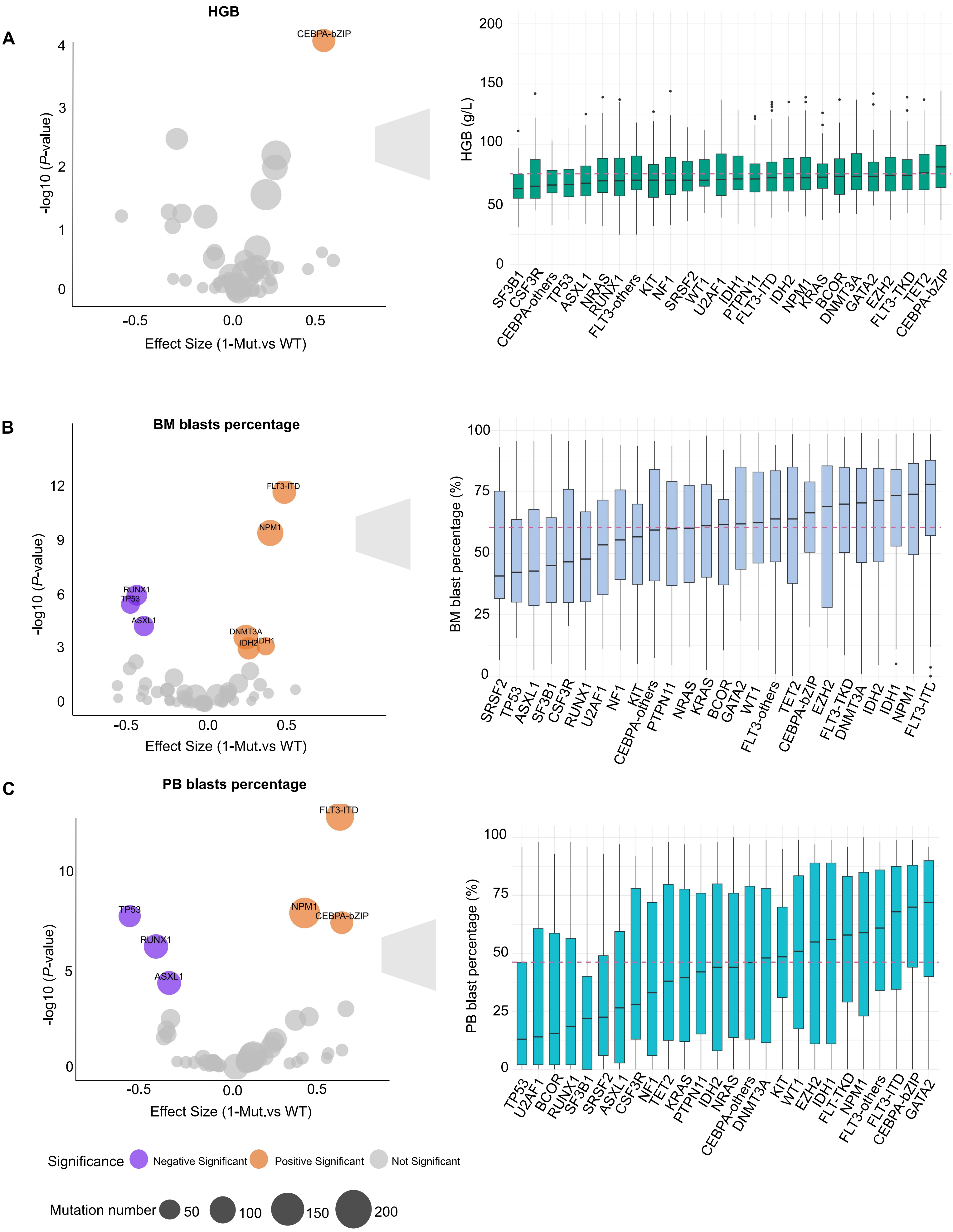
A
HGB
CEBPA-bZIP
-log10 (P-value)
Effect Size (1-Mut.vs WT)
HGB (g/L)
SF3B1
CSF3R
CEBPA-others
TP53
ASXL1
NRAS
RUNX1
FLT3-others
KIT
NF1
SRSF2
WT1
U2AF1
IDH1
PTPN11
FLT3-ITD
IDH2
NPM1
KRAS
BCOR
DNMT3A
GATA2
EZH2
FLT3-TKD
TET2
CEBPA-bZIP
B
BM blasts percentage
FLT3-ITD
NPM1
RUNX1
TP53
ASXL1
DNMT3A
IDH2
IDH1
-log10 (P-value)
Effect Size (1-Mut.vs WT)
BM blast percentage (%)
SRSF2
TP53
ASXL1
SF3B1
CSF3R
RUNX1
U2AF1
NF1
KIT
CEBPA-others
PTPN11
NRAS
KRAS
BCOR
GATA2
WT1
FLT3-others
TET2
CEBPA-bZIP
EZH2
FLT3-TKD
DNMT3A
IDH2
IDH1
NPM1
FLT3-ITD
C
PB blasts percentage
FLT3-ITD
TP53
NPM1
CEBPA-bZIP
RUNX1
ASXL1
-log10 (P-value)
Effect Size (1-Mut.vs WT)
PB blast percentage (%)
TP53
U2AF1
BCOR
RUNX1
SF3B1
SRSF2
ASXL1
CSF3R
NF1
TET2
KRAS
PTPN11
IDH2
NRAS
CEBPA-others
DNMT3A
KIT
WT1
EZH2
IDH1
FLT-TKD
NPM1
FLT3-others
FLT3-ITD
CEBPA-bZIP
GATA2
Significance
Negative Significant
Positive Significant
Not Significant
Mutation number
50
100
150
200

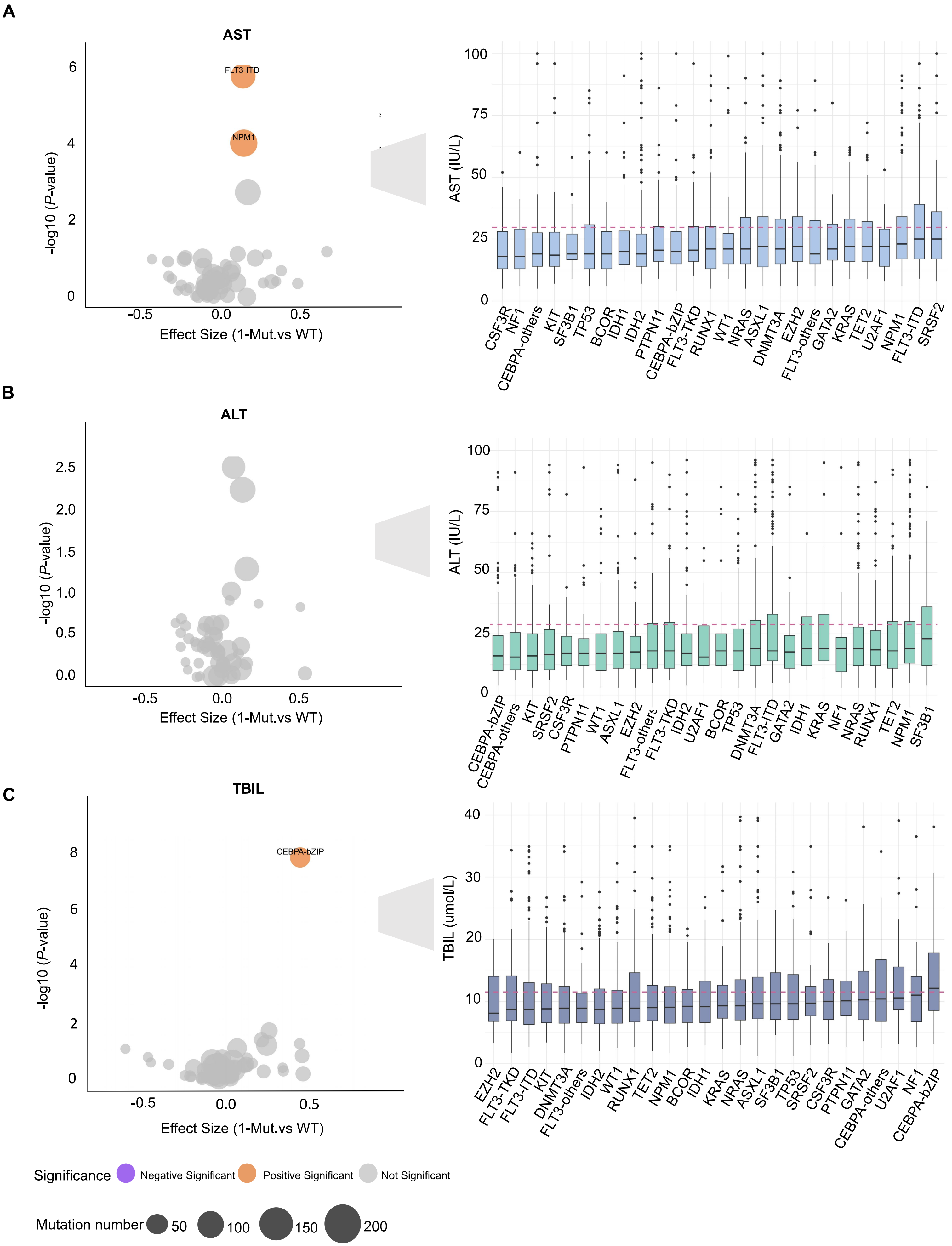

A
AST
FLT3-ITD
NPM1
-log10 (P-value)
Effect Size (1-Mut.vs WT)
AST (IU/L)
CSF3R NF1 CEBPA-others KIT SF3B1 TP53 BCOR IDH1 IDH2 PTPN11 CEBPA-bZIP FLT3-TKD RUNX1 WT1 NRAS ASXL1 DNMT3A EZH2 FLT3-others GATA2 KRAS TET2 U2AF1 NPM1 FLT3-ITD SRSF2
B
ALT
-log10 (P-value)
Effect Size (1-Mut.vs WT)
ALT (IU/L)
CEBPA-bZIP CEBPA-others KIT SRSF2 CSF3R PTPN11 WT1 ASXL1 EZH2 FLT3-others FLT3-TKD IDH2 U2AF1 BCOR TP53 DNMT3A FLT3-ITD GATA2 IDH1 KRAS NF1 NRAS RUNX1 TET2 NPM1 SF3B1
C
TBIL
CEBPA-bZIP
-log10 (P-value)
Effect Size (1-Mut.vs WT)
TBIL (umol/L)
EZH2 FLT3-TKD FLT3-ITD KIT DNMT3A FLT3-others IDH2 WT1 RUNX1 TET2 NPM1 BCOR IDH1 KRAS NRAS ASXL1 SF3B1 TP53 SRSF2 CSF3R PTPN11 GATA2 CEBPA-others U2AF1 NF1 CEBPA-bZIP
Significance
Negative Significant
Positive Significant
Not Significant
Mutation number
50
100
150
200

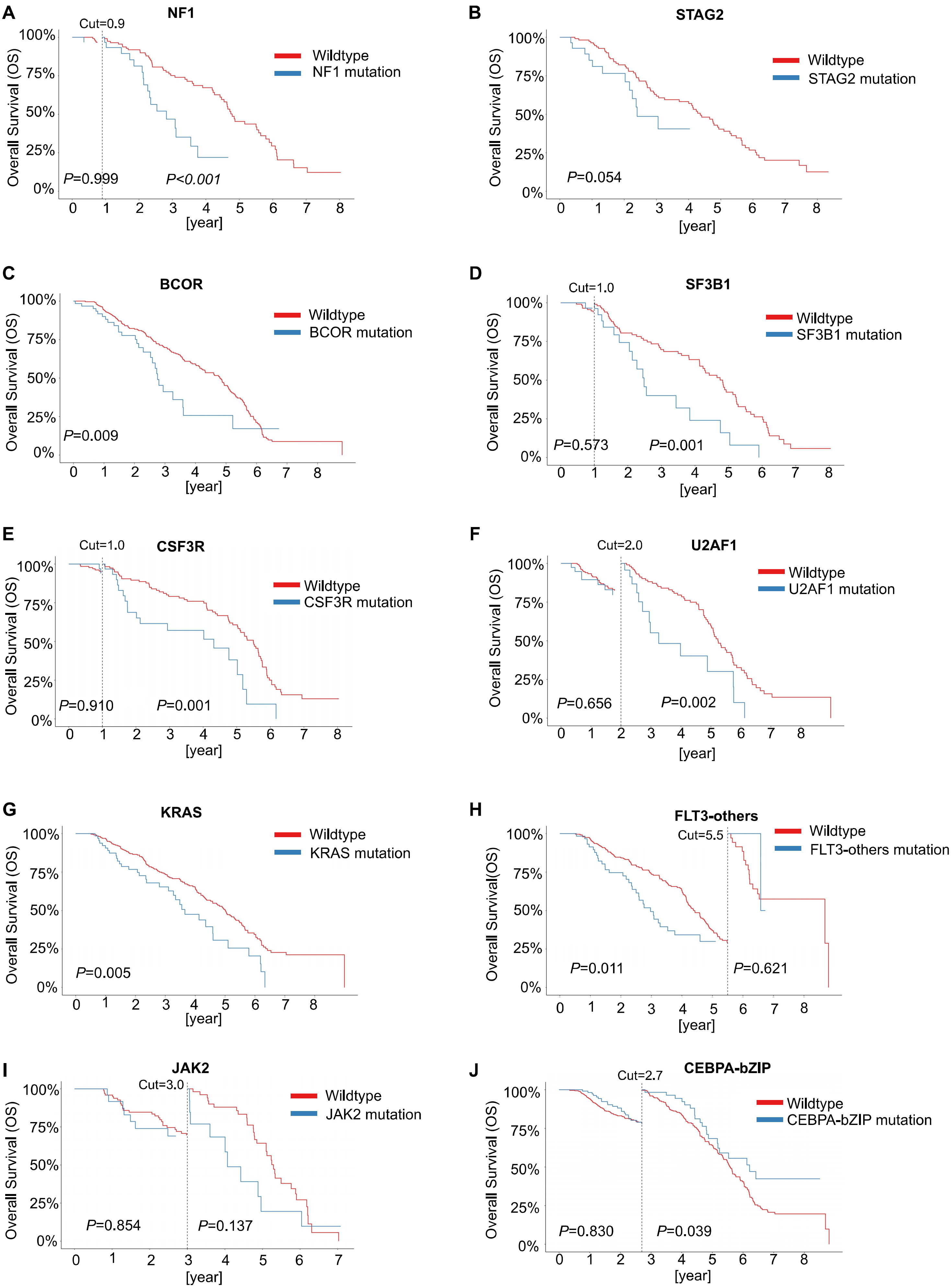

A
NF1
Cut=0.9
Wildtype
NF1 mutation
Overall Survival (OS)
100%
75%
50%
25%
0%
P=0.999
P<0.001
0 1 2 3 4 5 6 7 8
[year]
B
STAG2
Wildtype
STAG2 mutation
Overall Survival (OS)
100%
75%
50%
25%
0%
P=0.054
0 1 2 3 4 5 6 7 8
[year]
C
BCOR
Wildtype
BCOR mutation
Overall Survival (OS)
100%
75%
50%
25%
0%
P=0.009
0 1 2 3 4 5 6 7 8
[year]
D
SF3B1
Cut=1.0
Wildtype
SF3B1 mutation
Overall Survival (OS)
100%
75%
50%
25%
0%
P=0.573
P=0.001
0 1 2 3 4 5 6 7 8
[year]
E
CSF3R
Cut=1.0
Wildtype
CSF3R mutation
Overall Survival (OS)
100%
75%
50%
25%
0%
P=0.910
P=0.001
0 1 2 3 4 5 6 7 8
[year]
F
U2AF1
Cut=2.0
Wildtype
U2AF1 mutation
Overall Survival (OS)
100%
75%
50%
25%
0%
P=0.656
P=0.002
0 1 2 3 4 5 6 7 8
[year]
G
KRAS
Wildtype
KRAS mutation
Overall Survival (OS)
100%
75%
50%
25%
0%
P=0.005
0 1 2 3 4 5 6 7 8
[year]
H
FLT3-others
Cut=5.5
Wildtype
FLT3-others mutation
Overall Survival(OS)
100%
75%
50%
25%
0%
P=0.011
P=0.621
0 1 2 3 4 5 6 7 8
[year]
I
JAK2
Cut=3.0
Wildtype
JAK2 mutation
Overall Survival (OS)
100%
75%
50%
25%
0%
P=0.854
P=0.137
0 1 2 3 4 5 6 7
[year]
J
CEBPA-bZIP
Cut=2.7
Wildtype
CEBPA-bZIP mutation
Overall Survival (OS)
100%
75%
50%
25%
0%
P=0.830
P=0.039
0 1 2 3 4 5 6 7 8
[year]

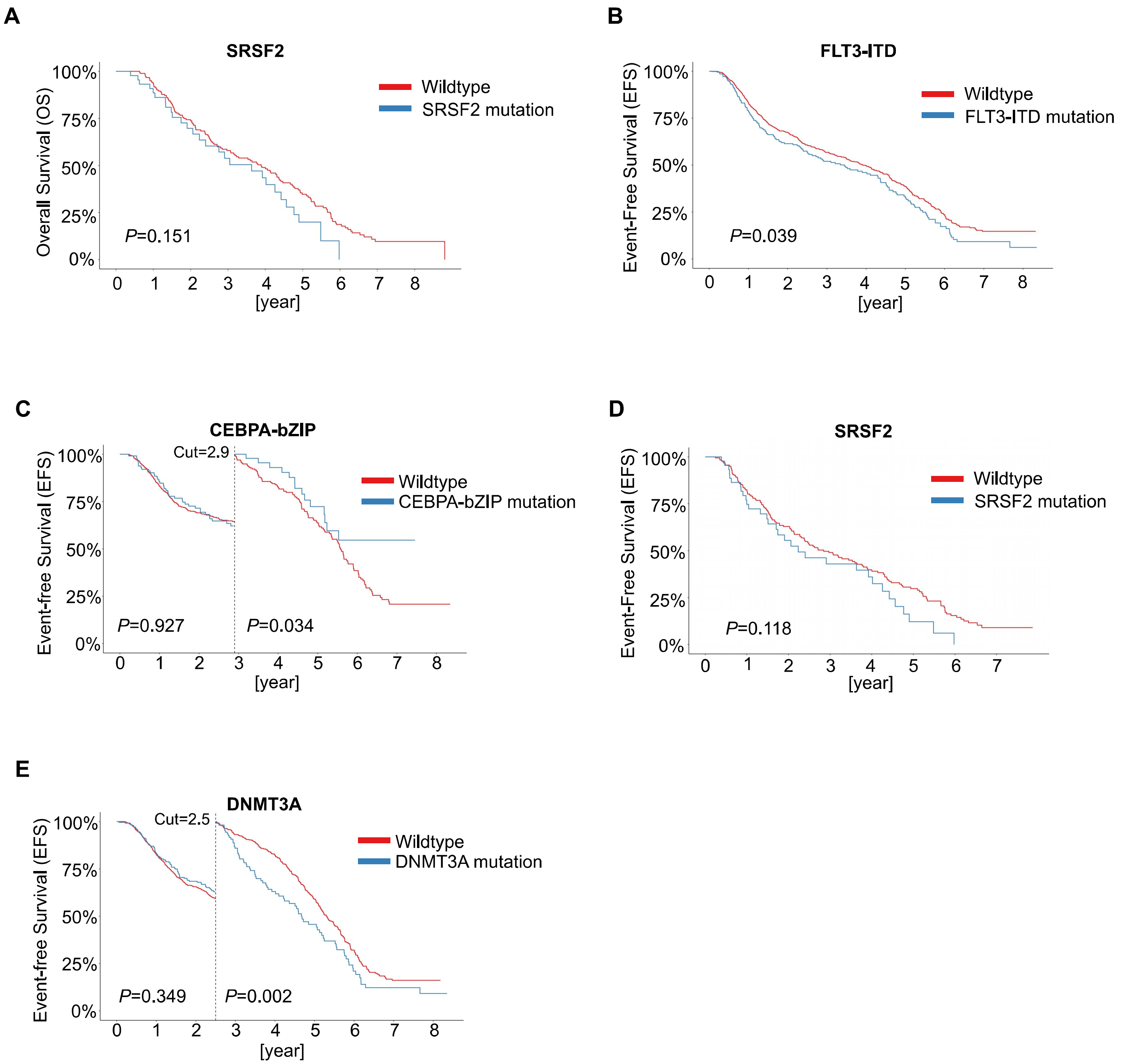
A
SRSF2
Wildtype
SRSF2 mutation
Overall Survival (OS)
100%
75%
50%
25%
0%
P=0.151
0 1 2 3 4 5 6 7 8
[year]
B
FLT3-ITD
Wildtype
FLT3-ITD mutation
Event-Free Survival (EFS)
100%
75%
50%
25%
0%
P=0.039
0 1 2 3 4 5 6 7 8
[year]
C
CEBPA-bZIP
Cut=2.9
Wildtype
CEBPA-bZIP mutation
Event-free Survival (EFS)
100%
75%
50%
25%
0%
P=0.927
P=0.034
0 1 2 3 4 5 6 7 8
[year]
D
SRSF2
Wildtype
SRSF2 mutation
Event-Free Survival (EFS)
100%
75%
50%
25%
0%
P=0.118
0 1 2 3 4 5 6 7
[year]
E
DNMT3A
Cut=2.5
Wildtype
DNMT3A mutation
Event-free Survival (EFS)
100%
75%
50%
25%
0%
P=0.349
P=0.002
0 1 2 3 4 5 6 7 8
[year]

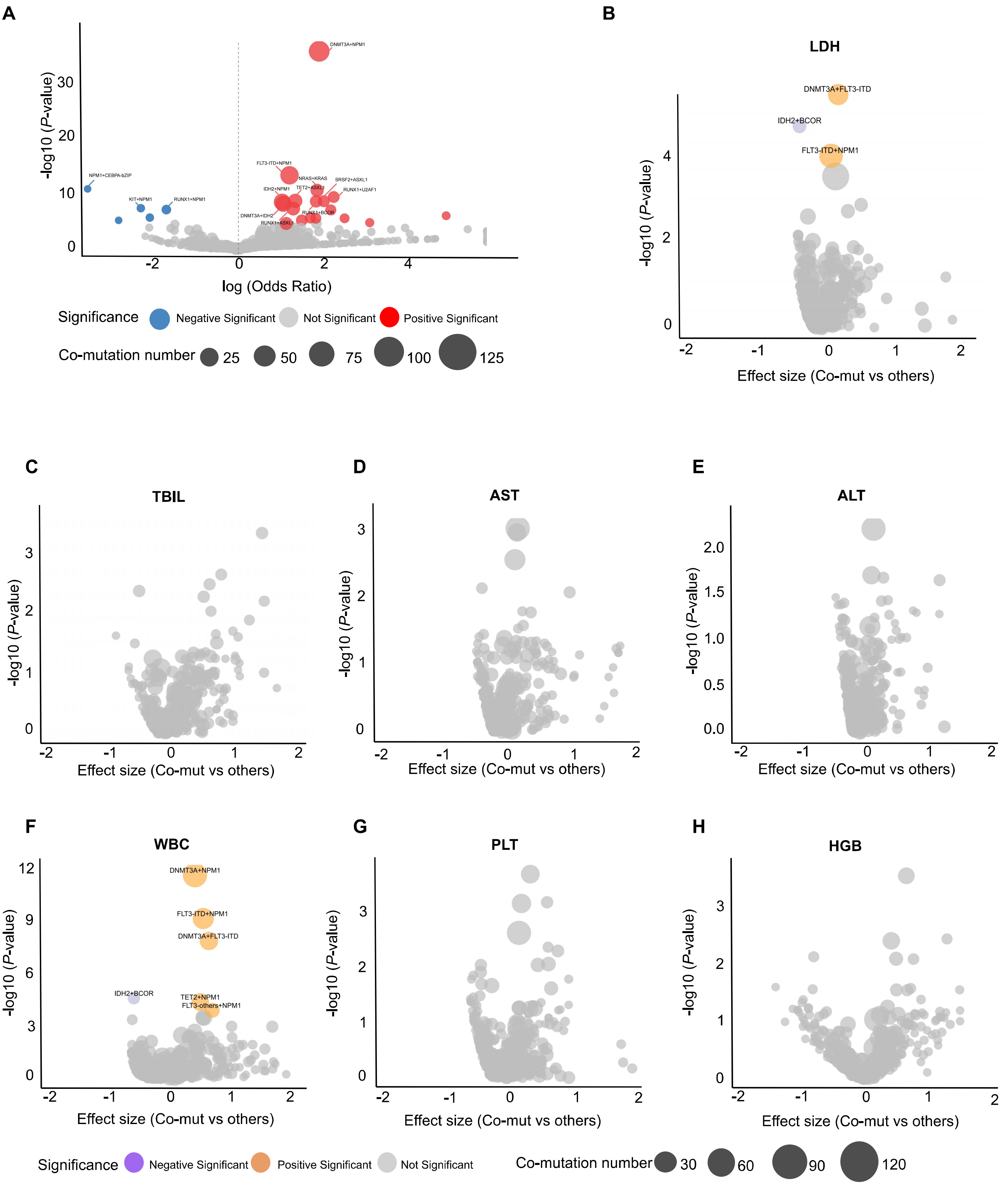

A
DNMT3A+NPM1
FLT3-ITD+NPM1
NRAS+KRAS
SRSF2+ASXL1
TET2+ASXL1
IDH2+NPM1
RUNX1+U2AF1
RUNX1+BCOR
DNMT3A+IDH2
RUNX1+ASXL1
NPM1+CEBPA-bZIP
KIT+NPM1
RUNX1+NPM1
-log10 (P-value)
log (Odds Ratio)
Significance
Negative Significant
Not Significant
Positive Significant
Co-mutation number
25
50
75
100
125
B
LDH
DNMT3A+FLT3-ITD
IDH2+BCOR
FLT3-ITD+NPM1
Effect size (Co-mut vs others)
C
TBIL
D
AST
E
ALT
F
WBC
DNMT3A+NPM1
FLT3-ITD+NPM1
DNMT3A+FLT3-ITD
IDH2+BCOR
TET2+NPM1
FLT3-others+NPM1
G
PLT
H
HGB
Significance
Negative Significant
Positive Significant
Not Significant
Co-mutation number
30
60
90
120
